# Potential of bioaugmentation of heavy metal contaminated soils in the Zambian Copperbelt using autochthonous filamentous fungi

**DOI:** 10.3389/fmicb.2022.1045671

**Published:** 2022-12-01

**Authors:** Leonce Dusengemungu, Cousins Gwanama, Grant Simuchimba, Benjamin Mubemba

**Affiliations:** ^1^School of Mathematics and Natural Sciences, The Copperbelt University, Kitwe, Zambia,; ^2^Africa Centre of Excellence for Sustainable Mining, The Copperbelt University, Kitwe, Zambia; ^3^School of Natural Resources, The Copperbelt University, Kitwe, Zambia

**Keywords:** bioaugmentation, *Aspergillus transmontanensis*, *Geotrichum candidum*, *Cladosporium*, heavy metal, contaminated soil

## Abstract

There is great potential to remediate heavy metal contaminated environments through bioaugmentation with filamentous fungi. However, these fungi have been poorly investigated in most developing countries, such as Zambia. Therefore, the present study aimed at isolating indigenous filamentous fungi from heavy metal contaminated soil and to explore their potential for use in bioaugmentation. The conventional streak plate method was used to isolate fungi from heavy metal-contaminated soil. Filamentous fungal isolates were identified using morphological and molecular techniques. The radial growth diameter technique was used to evaluate heavy metal tolerance of the fungi. The most abundant and highly tolerant fungi, identified as *Aspergillus transmontanensis*, *Cladosporium cladosporioides,* and *Geotrichum candidum* species, were used to bioremediate heavy metal contaminated soil samples with uncontaminated soil sample being employed as a control. A maximum tolerance index (TI) between 0.7 and 11.0 was observed for *A. transmontanensis*, and *G. candidum* while *C. cladosporioides* displayed the TI between 0.2 and 1.2 in the presence of 1,000 ppm of Cu, Co, Fe, Mn, and Zn. The interspecific interaction was analyzed to determine the compatibility among isolates. Our results showed mutual intermingling between the three evaluated fungal species, which confirms their common influence in biomineralization of heavy metals in contaminated soils. Maximum bio-removal capacities after 90 days were 72% for Cu, 99.8% for Co, 60.6% for Fe, 82.2% for Mn, and 100% for both Pb and Zn. This study has demonstrated the potential of highly resistant autochthonous fungal isolates to remediate the heavy metal contamination problem.

## Introduction

Soil bacteria, actinomycetes, and filamentous fungi are critical tools in bioremediation technologies. They are exploited due to their capacity to degrade organic and inorganic chemical contamination in soil and wastewater. During bioaugmentation, microbial systems are utilized to bioremediate-contaminated areas effectively. The positive outcome of bioaugmentation is the complete degradation of toxic substances, which are successfully transformed into carbon dioxide, cell biomass, and water, as observed previously (Gadd et al., 2014; Murugesan et al., 2014; Deshmukh et al., 2016; Gupta et al., 2017; Hassan et al., 2019, 2020). Microorganisms, principally bacteria and fungi, are popularly applied in the biodegradation of a wide range of petroleum compounds and bioaccumulation of toxic heavy metals (Mancera-López et al., 2008; Silva et al., 2009). However, previous studies have shown advantages of fungi over bacteria for the biodegradation, biomineralization, and storing toxic heavy metals in contaminated soils because fungal cell walls have high biosorption capacity for suspended solids as well as solutes (Tobin et al., 1994), while bacterial cell walls (gram negative bacteria) have shown high affinity only for aqueous metals (Chakravarty and Banerjee, 2012). For example, three indigenous fungi isolated from two aged and highly contaminated soils were able to grow in complex solid mixtures of high molecular weight hydrocarbons. *Rhizopus* sp.*, Penicillium funiculosum,* and *Aspergillus oryzae sydowii* removed 36, 30, and 17% of extra polycyclic aromatic hydrocarbons (PAH) respectively (Mancera-López et al., 2008). Similar results have been reported in bioremediation of PAH contaminated soil through bioaugmentation with filamentous fungi (*Penicillium* sp., *Penicillium Chyrsogenum*, *Ulocladium* sp., *Ulocladium atrum*, *Aspergillus terreus*, *Fusarium oxysporum*, and *Aspergillus parasiticus*; Wemedo and Aleruchi, 2020; Medaura et al., 2021). In addition, bioaugmentation with heavy metal-resistant filamentous fungi such as *Cladosporium* sp., *Didymella glomerata, Fusarium oxysporum*, *Phoma costaricensis*, and *Sarocladium kiliense* as bioremediators has been successful in removing mercury (Hg) from aqueous substrate (Văcar et al., 2021).

Due to a large reserve of copper (Cu) and cobalt (Co) minerals in the Copperbelt provinces of Democratic Republic of the Congo and in Zambia, increased mining activities have created environmental contamination and pollution resulting from the generation of large quantities of tailings overburden materials, waste rocks, and slags. These materials have increased heavy metal toxicity in agricultural land and raise serious concerns on the safety and quality of surface and groundwater systems (Pourret et al., 2015; Chileshe et al., 2019). The restoration of the post-mining landscape in Africa is currently dominated by phytoremediation. Several remediation successes have been reported in the limestone quarries in Kenya, the gold mine wasteland in Ghana, and sand mining tailings in South Africa (Tutu et al., 2008; Gathuru, 2011; Albert, 2015). However, the speed of post-mining landscape bioremediation practice is slow due to natural succession and revegetation processes (Festin et al., 2019).

Due to weathering effect, most landfill and mining waste materials from ongoing mining activities or decommissioned mines may result in heavy metal contamination of soil and water (Esshaimi et al., 2017). The use of soil and water heavily contaminated by Cu, Co, Pb, Zn, and Ni hinders plant growth due to the modifying effects of these elements on various vital enzymes responsible for different metabolic pathways (Pettersson and Ingri, 2001; Vítková et al., 2010; Khatri and Tyagi, 2015). In addition, previous studies have also demonstrated elevated heavy metals in plants grown near landfills (Da Opaluwa et al., 2012; Pehoiu et al., 2020). In Zambia, a study conducted to analyze the effect of heavy metal-polluted wastewater for irrigation at ‘New Farm’ in Mufulira, and Chilumba Gardens in Kafue demonstrated adverse effects on crop growth (Kapungwe, 2013).

Microorganisms play a significant role in soil health *via* biotransformation of heavy metals to more stable forms, biosorption, bioleaching, biomineralization, enzyme-catalyzed transformation and storing toxic elements (intracellular accumulation; Burford et al., 2003; Yu and Zhan, 2020; Zhang et al., 2022). Therefore, due to their rapid growth, low-cost culture media, and low-cost waste biomass availability, their immobilized biomass can be used for biosorption of heavy metal contaminants (Wu et al., 2021). Particular interest is in micromycetes because many species produce waste biomass that is available and affordable from the biotechnology industry (Velkova et al., 2018). In addition, many filamentous fungi have been investigated as alternative biosorbents for metal bioremoval and remarkable successes have been reported (Fawzy et al., 2017; Hassan et al., 2019). Fungi have a biogeochemical capacity to degrade pollutants and minimize the effect of these heavy metals on the environment (Bhandari and Bhatt, 2021). Besides, their mycelial network broadly enhances their degradative activity, making them a suitable bioremediation technology (Burford et al., 2003). Furthermore, fungi have negative charges on their cell wall surface due to anionic carboxyl and phosphate groups and positively charged amino groups, which determine the electrostatic attraction of metal ions upon formation of complexes with N or O donor atoms (Chitosan and Chitin; Das et al., 2007). These negative and positive charges greatly aid in their metal biosorption capacity (Tobin et al., 1994; Dusengemungu et al., 2021).

Nevertheless, more research is required to optimize the utilization of fungi to remove metal ions from polluted soil and water. Until recently, research into soil bioremediation with filamentous fungi focused primarily on their biodiversity and tolerance (Ahmad et al., 2012; Szada-Borzyszkowska et al., 2021). Even though fungi are understudied in Zambia, the country boasts a large variety of fungi, and the present study will contribute to the global comprehension of indigenous fungi. Furthermore, to add to the current knowledge about bioaugmentation potential, the study focused on supplementing a culture of fungi into soil contaminated with the heavy metals Cu and Co. Previously, supplementation of microorganisms into the natural or engineered environment has been used in trials on wastewater treatment processes and soil bioremediation, but practical application of these fungi is still lacking because they have not yet been optimized for large scale application (Singh et al., 2015; Bosco and Mollea, 2019). Diverse local mining environments harbour distinct plants and microorganisms, which may be useful for target specific bioremediation of heavy metal contaminants. Although tolerance and resistance of fungi to Cu and Co have been investigated widely, assessment regarding bioaugmentation with fungi in Cu- and Co-contaminated environments is still unclear (Dusengemungu et al., 2020). Therefore, screening established culture-collected strains and new isolates could contribute in identifying fungi strains that are able to biomineralize heavy metals more rapidly and efficiently. In this study, blended indigenous fungal isolates were used to investigate their potential in improving and speeding up the remediation of soil contaminated by heavy metals, particularly Cu and Co.

## Materials and methods

### Description of the sampling site

**Figure 1:**
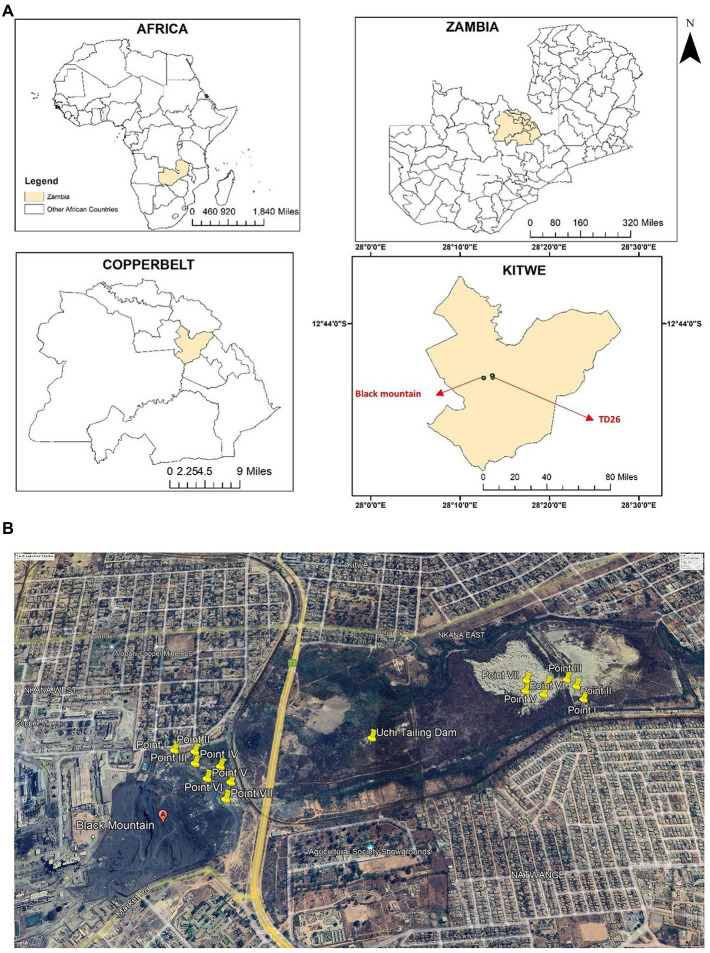
**(A)** The geographical location of the study sites, Kitwe district, in cream. The insert maps show Zambia’s global position and size, highlighting the Copperbelt Province in cream and Kitwe district in cream, showing the sampling location. Maps were generated using ArcGIS 10.5 (ESRI, Redland, California). **(B)** An aerial photograph shows yellow-coloured sampling points at Nkana tailing dam location (Black mountain), Uchi following dam location (TD26) and the surrounding area (Google Earth Satellite Image system, October 2022). The spatial sampling location was created using ArcMap 10 software (Environmental Systems Research Institute (ESRI), Redlands, CA, USA).

Two sites in Kitwe District of the Copperbelt Province of Zambia were selected for this study (Figure 1). The study sites were one Cu and Co mining waste dumpsite and one abandoned tailings dam. Nkana Slag Dumpsite, also known as the “Black Mountain, “is located at latitude 12^o^50’ S and longitude 28^o^12′ E, while Uchi Tailing Dam (TD26) is located at latitude 12^o^49′ S and longitude 28^o^13′ E. From 1931 to 2009, mining waste from the copper smelter was dumped in what later became the Black Mountain. It is estimated to contain more than 30 million metric tonnes of smelter slag containing 0.34 to 4.5% Co and about 1.2% Cu (Ettler et al., 2011). As of August 2022, the black mountain is being cleared for further ore extraction and processing using more recent and efficient technologies. The Uchi Tailing Dam (TD26) is one of the abandoned tailing dams used by Nkana Mine from 1931 until 2009 to deposit the remains of Co extraction. The Black Mountain and Uchi Tailing Dam are located between current residential and former agricultural areas. These sampling sites have been previously described (Dusengemungu et al., 2022).

### Analytical procedure and collection of mining waste soil from the tailing dams

Soil samples were collected from a 0–20 cm depth (‘topsoil’) using a trowel and were homogenized. Seven samples were collected from each dumpsite and stored in a polythene bag (Figures 1A,B) as previously done (Dusengemungu et al., 2022). The samples were analysed for pH and concentration of six heavy metals. For heavy metal analysis, soil samples were ground with a wooden mortar, air-dried for 48 h, and sieved through a 2-mm mesh. Samples were then extracted using acid digestion (Cook et al., 1997). Thereafter, they were analysed for Cu, Co, Fe, Mn, Pb, and Zn using the Atomic Absorption Spectrophotometry (AAS) PinAAcle 900H in a commercial lab at Sable Zinc Mines in a town called Kabwe. Each sample was digested and analysed three times to the improve the consistency in the results.

**Figure 2:**
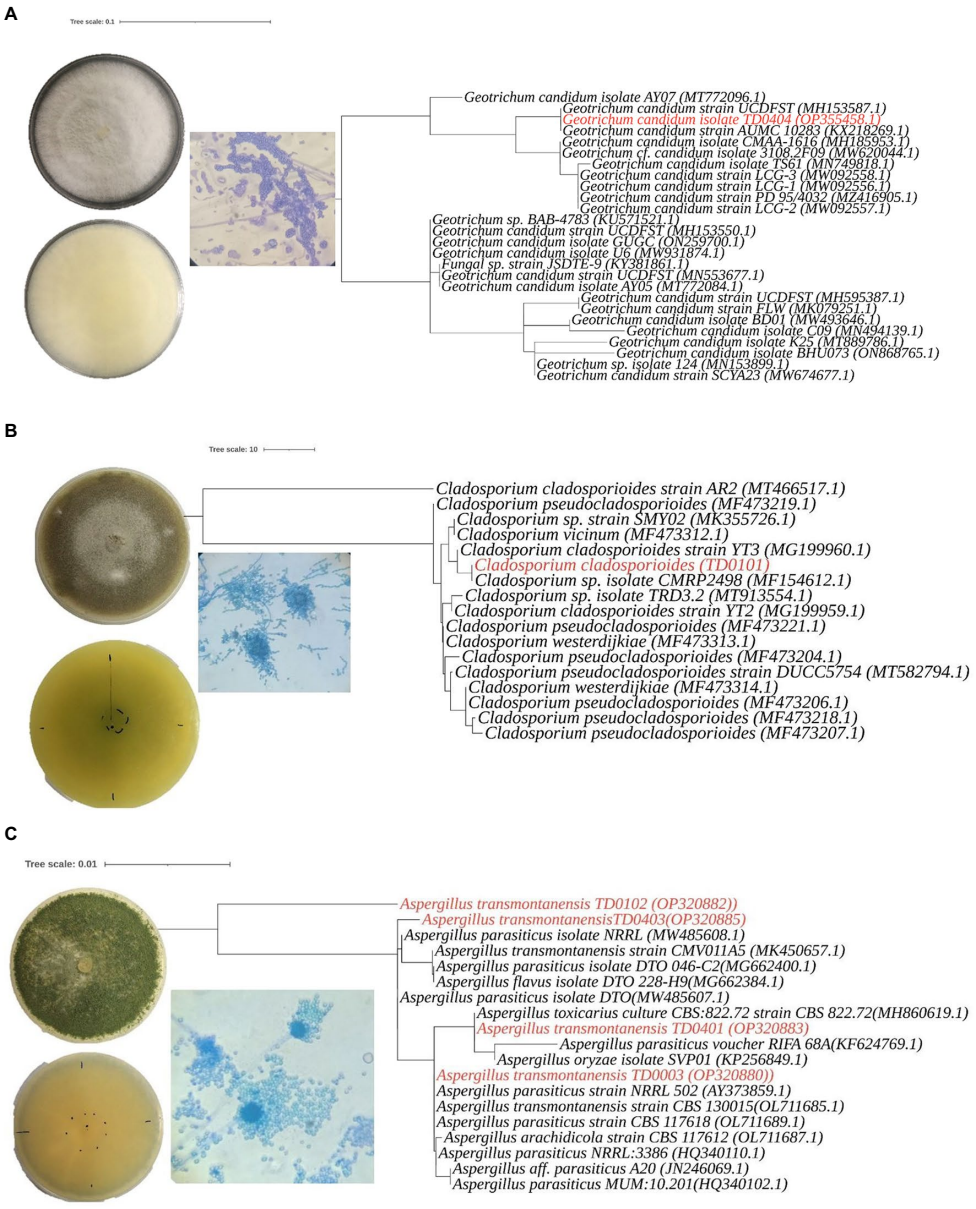
Morphological characteristics andphylogenetic analysis of three *Aspergillus transmontanensis*, *Cladosporium cladosporioides*, and *Geotrichum candidum* isolated from the contaminated soils of Nkana Slag Dump and TD 26. Morphology of *Geotrichum candidum* **(A)**, *Cladosporium cladosporioides* **(B)**, *Aspergillus transmontanensis* **(C)**, on PDA medium. Light microscopy of conidiophores, conidia of the fungal isolates on PDA medium (400 x; **A– C**).

### Isolation and morphological characterization of fungi

Distilled water was used for serial dilution to isolate filamentous fungi from soil samples. Firstly, 1 g of dumpsite soil was dissolved in 10 ml of sterile distilled water and agitated on a rotary shaker for about 20 min at 220 rpm. Afterwards, solid particles were allowed to settle for 30 min to allow for serial dilutions that ranged between 10^−1^ to 10^−7^. From each dilution, 0.1 ml was pipetted onto Potato Dextrose Agar (PDA) medium (Hi Media Laboratories Pvt. Ltd. Mumbai, India) and then spread homogenously using a sterile spreader, followed by incubation at room temperature for 7 days. Resulting mixed fungi colonies were sub-cultured on PDA slants and incubated at room temperature for 7 days to isolate pure cultures. Morphological identification of fungi was done based on previously standardised methodologies of Gente et al. (2006), Schubert et al. (2007), Samson et al. (2014). The pure cultures were characterized macroscopically by observing morphological characteristics such as colony colour, presence or absence of aerial hyphae, the quantity of aerial hyphae, colony surface texture, colony margin, pattern, and pigment exuded. Wet mounts of the fungal isolates were then prepared using the lactophenol cotton blue technique (Leck, 1999). The slides were viewed under a microscope (×40) to identify the isolates for the presence of spores, the nature of columella phialides, conidiospore colour, etc. (Yin et al., 2017). Most isolates were tentatively identified to the species level using the standard identification keys (Watanabe, 2016).

### Molecular identification and characterization of fungi

For molecular identification, fungal DNA was extracted from the cultures using the Quick-DNA™ Fungal/Bacterial Miniprep Kit (Zymo Research, Irvine, USA). Thereafter, the internal transcribed spacer (ITS) region was amplified using OneTaq® Quick-Load® 2X Master Mix (New England Biolabs Inc., Ipswich, UK) with primers ITS1 (5’TCCGTAGGTGAACCTGCGG3´) and ITS 4 (5’TCCTCCGCTTATTGATATGC3´) as previously reported (Gherbawy and Voigt, 2010). The resulting PCR products were run on 1.2% agarose gel, and purified using the Zymoclean™ Gel DNA Recovery Kit (Zymo Research, Irvine, USA). The purified fragments were then sequenced forward and reverse using the Nimagen, BrilliantDye™ Terminator Cycle Sequencing Kit V3.1 (ThermoFisher Scientific, Massachusetts, USA) and purified using the Zymo Research ZR-96 DNA Sequencing Clean-up Kit™ (Irvine, USA). The purified fragments were analyzed on the ABI 3500xl Genetic Analyzer (Applied Biosystems, ThermoFisher Scientific, Massachusetts, USA). CLC Bio Main Workbench v7.6 (QIAGEN, Venlo, Netherlands) was used to analyze and assemble the ab1 sequence files generated by the ABI 3500XL Genetic Analyzer. Thereafter, a BLAST search was conducted to identify the sequenced fungal species. Sequences obtained in the present study were deposited in GeneBank under accession numbers OP320880, OP320881, OP320882, OP320883, OP320884, OP320885, and OP355458 (Table 1).

**Table 1:** Autochthonous fungi isolates used in this study.

Strain number	Source	GeneBank accession number	Species name
TD0003	¨Black mountain, and Uchi tailing dam (TD26)¨—Copperbelt, Kitwe, Zambia	OP320880	*Aspergillus transmontanensis*
TD0101	¨Black mountain, and Uchi tailing dam (TD26)¨—Copperbelt, Kitwe, Zambia	OP320881	*Cladosporium cladosporioides*
TD0102	¨Black mountain, and Uchi tailing dam (TD26)¨—Copperbelt, Kitwe, Zambia	OP320882	*Aspergillus transmontanensis*
TD0401	¨Black mountain, and Uchi tailing dam (TD26)¨—Copperbelt, Kitwe, Zambia	OP320883	*Aspergillus transmontanensis*
TD0403	¨Black mountain, and Uchi tailing dam (TD26)¨—Copperbelt, Kitwe, Zambia	OP320885	*Aspergillus transmontanensis*
TD0404	¨Black mountain, and Uchi tailing dam (TD26)¨—Copperbelt, Kitwe, Zambia	OP355458	*Geotrichum candidum*

To further understand how fungal species identified in the present study were related to one another and their relationships to other closely related species, multiple sequence alignment was performed using the FFT-NS-2 algorithm available in the multiple sequence alignment programme (MAFFT) set to default settings^1^ (Katoh et al., 2019). The resulting alignment was then used to construct a maximum likelihood (ML) phylogenetic tree using the PhyML Online server^2^ (Guindon et al., 2005). Tree construction employed the smart model selection (SMS; Lefort et al., 2017) and the Bayesian Information Criterion using default settings. Branch robustness was estimated using the Shimodaira-Hasegawa-like approximate likelihood ratio test (SH-aLRT). The best-fitting root of the resulting phylogeny was estimated using the heuristic residual mean squared function, aimed at minimizing the variance of root-to-tip distances using TempEst v1.5.3 (Rambaut et al., 2016). Finally, the tree file was edited using Interactive Tree of Life (iTOL) v5, an online tool for phylogenetic tree display and annotation (Letunic and Bork, 2021).

### Determination of metal tolerance of fungi

The methods used by Lobos et al. (2020) were employed to determine the tolerance capacity of filamentous fungi. Heavy metals stock solutions were obtained by diluting the metal salts CuSO_4_.5H_2_O, CoCl_2_, FeSO4.7H_2_O, MnCl_2_.6H_2_O, and ZnSO_4_.7H_2_O with distilled water in a conical flask, followed by serial dilutions to obtain 500, 1,000, 2000, and 5,000 ppm concentrations. No tolerance test was performed with Pb due to the low lead concentration in our sample soil. The sub-cultured fungal isolates were transferred onto PDA containing different metal concentrations and incubated for 7 days at room temperature. Fungal radial growth was measured as the diameter of the expanding mycelium on day 3 and day 7, respectively. The tolerance index was calculated as the ratio of the radial growth of the metal amended media to that of the radial growth of the untreated control (Equation 1). The maximum tolerance index, T*i*, was calculated as:


Ti=Dt/Du

D*t* is the treated plates’ mycelial diameter, and D*u* is the mycelial diameter of untreated plates (in mm; Equation 1; Coelho et al., 2020).

### Microbial formulation and soil mycoremediation

#### Paired interactions in PDA media

In order to use the fungal species as a consortium, there was need to test their compatibility. To achieve this, we tested the kinetic growth rate of the three isolates by inoculating pairs of isolates onto PDA media. Two-day old mycelium was used because it had not yet sporulated and therefore not prone to cause spore dispersion. A 5 mm disc of two-day-old mycelium of each isolate was placed across each other on the same plate. All the three possible isolate combinations were tested. Fungal growth and macroscopic morphology characteristic were observed daily. The measurement of colony growth was carried out on day 3, 7 and 28 and stopped when the mycelial growth reached the edges of the Petri dishes (in most cases by day 28). The fungal growth rate was classified as slow (0–1 mm/d), moderate (1–3 mm/d), moderate (1–3 mm/d), or fast (3–6 mm/d).

#### Soil mycoremediation

Fungal isolates that demonstrated tolerance to high metal concentrations were employed for subsequent bioaugmentation experiments. Pure fungal strains were grown on PDA plates for 7 days at room temperature. The Erlenmeyer flasks containing potato dextrose broth (PDB) were inoculated with three 5 mm disc of 7-day-old mycelia and incubated at room temperature while shaking at 150 rpm on a rotary shaker. An equal volume of the pure strain fungal inoculums from each broth culture was drawn and mixed to form the bioaugmentation blend based on paired interactions tests and the isolates growth in PDB. The 250 ml blended, highly tolerant fungi were diluted with 250 ml distilled water. For five consecutive days, 100 ml of the bioaugmentation blend was added and mixed with the contaminated soil, initiating the bioremediation process for 20 samples, each containing two to four kilograms of soil. Treatment were kept for 90 days. Holes were made in polybags to drain excess water and aerate the mixture of soil and microorganisms. The soil moisture content was held constant through regular watering with distilled water. Soil subsamples were collected on days 1, 30 and 90 to determine pH and metal concentrations. The pH of soil samples was measured using a combined electrodes pH/EC meter (Multi 3320_Xylem Analytics, Weilheim, Germany). The mining waste soil was also examined for Cu, Co, Fe, Mn, Pb, and Zn using an Atomic Absorption Spectrophotometer (AAS) equipped with SyngistixTM for AA software, version 4.0, at a commercial lab at Sable Zinc Mines in Kabwe as described (Emenike et al., 2017; Khan et al., 2019a).

The heavy metal bioremoval capacity was assessed using the equation below as described by Hassan et al. (2019).


$$\%ofheavymetalremoval=\frac{C_{0\left(x\right)}-C_{F\left(X\right)}}{C_{0\left(x\right)}}\times 100\%$$

Where:

$C_{0\left(x\right)}$ = initial concentration of metal “x” in the soil mine waste at the beginning of the experiment.

$C_{F\left(x\right)}$= final concentration of metal “x” in the soil at the end of the experiment.

### Statistical analysis

We used R Version 4.1.0 (R Core Team, 2020) on R studio version 1.4.1717 (Rstudio Inc., Boston, MA, USA; RStudio, 2016) for data analysis. The multiple (pairwise comparison) test was applied to determine significant differences in mean heavy metal concentrations of different dumpsites. Using the stats package, the pairwise comparison test was performed (R Core Team, 2020). The gglot2 package (Wickham, 2009) was also used to create a box plot of heavy metal data.

## Results

### Identity and phylogenetic patterns of isolated fungi

The most abundant, diverse and culturable species from the study sites in Kitwe were *Geotrichum candidum*, *Aspergillus* sp. and *Cladosporium* sp. *Cladosporium* sp. recovered had a slow growth rate compared with other autochthonous fungi from the same location. In addition, they were less diverse. All the isolates recovered from the site belonged to the phylum Ascomycota (Table 1).

Molecular identification was performed to efficiently distinguish the isolated fungi than was possible by morphological characterization alone. This was especially the case for the *Aspergillus* sp., where the isolates had identical macro- and microscopic traits (production of arthrospores). In the molecular characterization, a single pattern band around 374 to 752 bp amplified from the ITS region was used to identify the isolates. Based on the BLAST search and phylogenetic placement, isolates TD0003, TD0102, TD0401, and TD0403 were determined to be *Aspergillus transmontanensis* and had a percentage similarity of 99.83, 97.16, 99.67, and 99.83%, respectively to the closest *Aspergillus transmontanensis* strain with GenBank accession number MK450657 isolated from the soil in Zambia. A close phylogenetic relationship was also observed with *A. parasiticus* though a 100% query cover was with *A. transmontanensis.* Therefore, isolates TD0102 (OP20882), TD0403 (OP320885), TD0401 (OP320883) and (TD0003) (OP320880) from the present study were determined to belong to the Aspergillus spp (Figure 2).

Phylogenetic placement for TD0101 which was morphologically identified as *Cladosporium* sp. revealed that it was indeed a *Cladosporium* sp. with 100% query cover and 99.82% similarity *Cladosporium cladosporioides* (with the GenBank accession number OM415957.1)*. Cladosporium cladosporioides* isolated in this study was also closely related to the *Cladosporium* sp. with accession number MT913554.1isolated from heavy metal contaminated soils in Turda, Cluy County, Romania (Văcar et al., 2021). The same strain was also recovered from arable soils elsewhere (Moll et al., 2016; Figure 2). The isolate TD0404 morphologically identified as *Geotrichum candidum* was confirmed with 100% query cover and 100% similarity to *Geotrichum candidum.* Both blast and phylogenetic analyse showed that the sequence data for the ITS region for *Geotrichum candidum* (OP355458) sequenced in this study shares 100% 1. Similarity with the Fungal strain JSDTE-9 with the GeneBank accession number KY381861, which was isolated from *Sophora tonkinesis* gagnep seeds (Liang et al., 2022) and it also shares 100% similarity with *Geotrichum candidum* reported for sour-rot of melon in Brazil with the Genebank accession number MH185953 (Halfeld-Vieira et al., 2015).

Considering the diverse composition of the Nkana Slag Dump and Uchi Tailing Dam, the predominance of these fungi groups in the contaminated soil attests to their exceptional ability to survive in adverse conditions.

### Fungal heavy metal tolerance capacity

**Figure 3:**
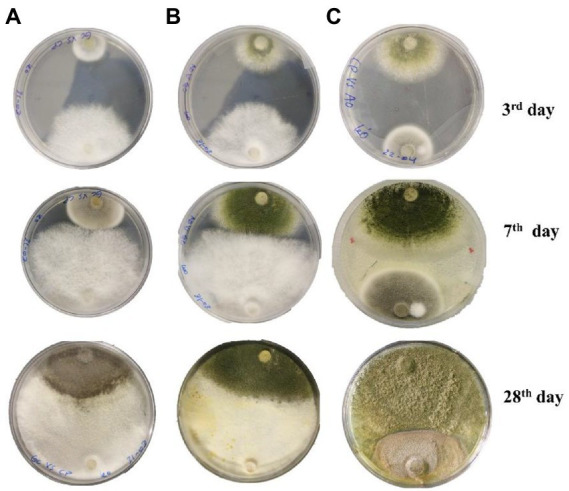
Interspecific interaction outcome of soil fungi grown on PDA media. **(A)** *Cladosporium cladosporioides* vs. *Geotrichum candidum*, **(B)** *Geotrichum candidum* vs. *Aspergillus transmontanensis,* **(C)** *Aspergillus transmontanensis* vs. *Cladosporium cladosporioides.*

Mycelia radial growth response of *A. transmontanensis, C. Cladosporioides* and *G. candidum* spp. to the diverse concentration of Cu and Co varied among the species (Figure 3). The exposure of *A. transmontanensis* species to high Cu and Co concentrations (>5,000 ppm) inhibited growth, whereby the radial growth of mycelia was significantly shorter with tolerance indices (T*i*) below 0.5 in comparison with the control (Table 2). The exposure of *G. candidum* and *C. cladosporioides* to a higher concentration of Cu and Co significantly limited the mycelial radial growth, with the tolerance indices (T*i*) below 0.2 compared to the control (Table 2). Tolerance indices of *G. candidum* and *C. cladosporioides* after exposure to 2,000 to 5,000 ppm of both Cu and Co were lower than for the control. Overall, the mycelial radial growth of *A. transmontanensis* fungal isolates in Cu and Co enriched media was relatively higher in all concentrations (500, 1,000, 2,000, and 5,000 ppm), as is shown by the very high tolerance index values of 1–0.83 in 500, 0.2–1.2 in 1,000 ppm Cu, Co and 0.6–0.38 in 2,000–5,000 ppm Cu, Co. However, the other *C. cladosporioides* tolerance index against high concentrations of Cu and Co varied between 0.44 and 0.001. Therefore, *C. cladosporioides* was considered to be less tolerant in comparison with *G. candidum*, which had a tolerance index that ranged between 0.875 and 0.02.

**Table 2:** Tolerance index levels of isolated fungal strains in heavy metal supplemented media.

Heavy metals	Fungi isolate	Concentrations (ppm)				The highest metal concentration (ppm) tolerated in PDA	“World permissible limit in soil (ppm)” ^c^
Cu		500	1,000	2,000	5,000		38.9
	*Aspergillus transmontanensis^a^*	1	1	0.8	0.5–0.38	5,000	
	*Geotrichum candidum^a^*	1	1	1	0.125	5,000	
	*Cladosporium cladosporioides^b^*	0.4	0.2	0.2	0	2,000	
Co			1,000	2,000	5,000		13
	*Aspergillus transmontanensis^a^*	1	1–0.83	0.44–0.38	0.22	5,000	
	*Geotrichum candidum^a^*	1	0.7	0.3	0.01	2,000	
	*Cladosporium cladosporioides^b^*	0.3	0.22–0.24	0.1	0.01	1,000	
Fe			1,000	2,000	5,000		29,400
	*Aspergillus transmontanensis^a^*	1	1	1	1	8,000	
	*Geotrichum candidum^a^*	1.2	1.113	1.132	0.377	8,000	
	*Cladosporium cladosporioides^b^*	1.4	1.219	0.902	0.487	4,000	
Mn			1,000	2,000	5,000		580
	*Aspergillus transmontanensis^a^*	1	1	0.89	0.57	5,000	
	*Geotrichum candidum^a^*	1	0.789	0.736	0.694	4,000	
	*Cladosporium cladosporioides^b^*	1	1	1	1	5,000	
Zn			1,000	2,000	5,000		74
	*Aspergillus transmontanensis^a^*	1	1	1	1	6,000	
	*Geotrichum candidum^a^*	1	0.76	0.56	0.15	6,000	
	*Cladosporium cladosporioides^b^*	1	0.63	0.31	0.21	5,000	
	Tolerance index value:						
	0.00–0.39 (very low tolerance)						
	0.40–0.59 (low tolerance)						
	0.60–0.79 (moderate tolerance),						
	0.80–0.99 (high tolerance)						
	1.00 > (very high tolerance).						


The letter (a) is for highly tolerant isolates and (b) for moderately tolerant isolate. (c) Staniland et al. (2010) and Oladipo et al. (2018).

A large disparity in Fe and Mn tolerance was noticed compared to other metals employed in the study. All the isolates showed high tolerance to extreme concentrations of Fe and Mn. The same findings were reported by Anahid et al. (2011). *A. transmontanensis* was recognized as highly tolerant to Zn. However, *G. candidum* and *Cladosporium cladosporioides* were less tolerant to the high concentration of Zn.

### *In vitro* interaction of fungal isolates

During *in vitro* co-incubation of the three fungal isolates, similar interaction patterns, namely mutual intermingling, were observed for the combinations of *C. cladosporioides* vs. *G. candidum, A. transmontanensis vs* G*. candidum* and *C. cladosporioides* vs. *A. transmontanensis.* In mutual intermingling, both isolates progressed unrestrained and developed in each other’s space (Figure 3). However, after maximum incubation days, *G. candidum* showed high competition for space against *C. cladosporioides* and *A. transmontanensis* while *A. transmontanensis* grew over and around *C. cladosporioides.*

### Heavy metal bioremoval by fungi

#### The pH of contaminated and amended soil

**Figure 4:**
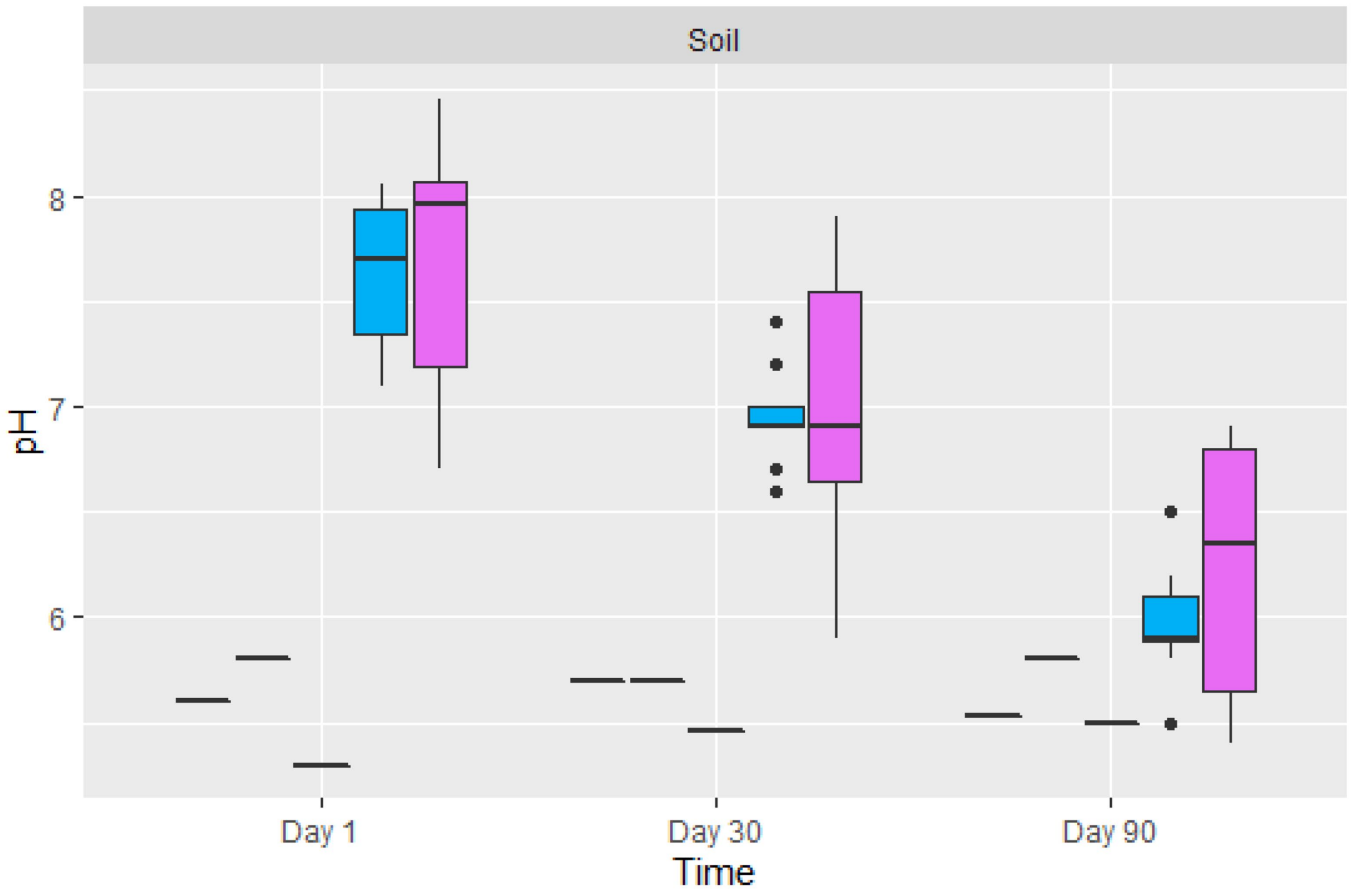
The boxplot of the average pH value of control (black lines) and two sites amended soil samples (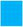 TD26: and 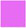 “BM”, the black mountain). The solid black lines inside the boxplot represent the median value, and the black dots represent the individual soil samples.

The average pH of the contaminated soil from the Nkana Slag Dump varied between 7.1 and 8.05, while the pH at TD 26 varied between 6.7 and 8.4 on the initial day of the experiment (Figure 4). Fungal treatment reduced the pH of soils for both sites to 5.4 and 6.9, respectively. The control soil samples maintained their pH between 5.3 and 5.8. The average pH of the treated soil sample (“BM”, the black mountain and TD26) reduced significantly after day 90 of the treatment.

#### Fungal heavy metal removal

**Figure 5:**
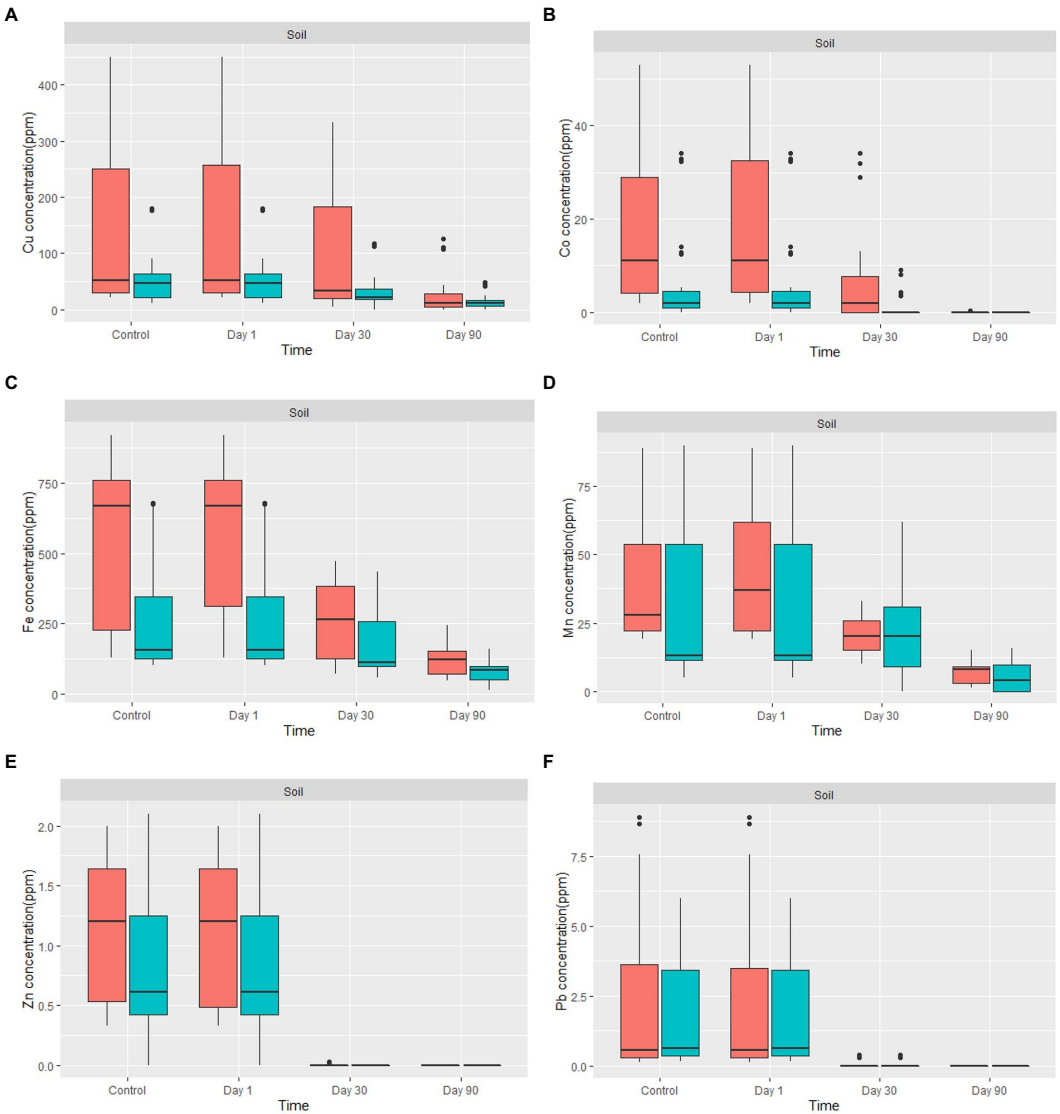
The boxplot shows **(A)** Cu, **(B)** Co, **(C)** Fe, **(D)** Mn, **(E)** Zn, and **(F)** Pb concentration for *in-situ* mycoremediation of metal contaminated soil for 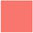 Nkana Slag Dump and 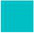 TD 26. The solid black lines inside the boxplot represent the median value, and the black dots represent the individual soil samples.

The amount of metal removed increased as the incubation period progressed (Figure 5). Fungal-treated soil had significantly reduced heavy metals content in comparison to non-treated soil. For example, the amount of Cu % removed by the fungal biomasses after 90 days of treatment was between 33 and 100%. The average removal of Cu from all treated contaminated soil was 72.08%. Due to the low concentration level of Co in the treated soil, the percentage removal of Co after 30 days of treatment was 100%, except for two samples where the removal was 39.45 and 41.9% after 30 days. After 90 days of treatment, Co was 100% removed from all our soil samples. Fe content in the soil of Nkana Slag Dump and Uchi Tailing Dams was exceptionally high, initially. Between 32 and 90.5% of the Fe, was removed. The overall Fe removal from all samples was 60%. For Mn, the amount removed after 90 days of treatment ranged between 55.7 and 100%. The average removal of Mn from all treated soil after 90 days was 82%. Soil samples from both study sites had negligibly low Pb and Zn content. It was observed that after only 30 days of treatment, Pb and Zn were not detectable. Comparatively, the maximum removal capacity of Co was more significant than the other studied metals.

## Discussion

### Identity and phylogenetic patterns of isolated fungi

Previous studies have reported a broad range of autochtonous saprotrophic microfungi exhibiting astounding levels of resistance to heavy metals. These included as *Aspergillus* sp., *Trichoderma* sp., *Penicillium* sp., *Geotrichum* sp., *Cladosporium* sp. (Lestan et al., 1996; Zafar, 2007; Ren et al., 2009; Joshi et al., 2011; Alori and Fawole, 2012; Hassan et al., 2020; Sey and Belford, 2021). Their potential for bioremediation has been investigated recently, including their capability to assist with phytoremediation of heavy metal-contaminated soil and their capacity to remove these metals from single or multi-metal solutions or contaminated soil bioaugmented with fungal consortia (Coelho et al., 2020; Dusengemungu et al., 2020; Hassan et al., 2020).

However, these fungal communities are target specific, so they cannot be utilized to bioremediate all types of contaminants unless they are trained (Singh and Roy, 2021). Our study has isolated similar fungi commonly reported for bioremediation purposes. Compared with sizeable fungal biodiversity reported elsewhere in a heavy metal contaminated environment, it is suggested that only a small fraction of fungal diversity in the study area has been revealed. To our knowledge, this is the first report of filamentous fungi isolated from highly Cu and Co-contaminated environments in Zambia and have shown potential for remove Cu, Co, Mn, Fe, Zn and Pb.

*Aspergillus transmontanensis* in this study shares close similarity to a diverse range of *A. transmontanensis* and *A. parasiticus* isolated from a wide range of sources (Visagie and Houbraken, 2020). The *Aspergillus* species are filamentous fungi which are ubiquitous in the environment (Gherbawy and Voigt, 2010). They possess many industrial applications ranging from harmful to beneficial. Beneficial applications include practical uses in the biosorption of heavy metals from contaminated sites. Among the popular species of *Aspergillus* described as heavy metal tolerant and used for biosorption are *A. niger, A. flavus, A. versicolor, and A. tamarii* (Simate et al., 2010; Hansda et al., 2016; Oladipo et al., 2018) These species have an enormous ability to create a metal sink, coupled with their capacity to produce organic acids that can bioleach metals.

Previous reports have classified *A. transmontanensis* as fungi of the *Trichocomaceae* family (Lee and Yamamoto, 2015). *Aspergillus transmontanensis* is closely similar to *A. parasiticus*, but it mainly possesses biseriate conidial heads. *A. parasiticus* usually has primarily uniseriate conidial heads, and *A. transmontanensis* produces larger abundant brown sclerotia than *A. parasiticus* (Moretti, 2017). It appears that *A. transmontanensis* is less widespread than *A. parasiticus* and that they are both better adapted to surviving in the soil and less reliant on crop infection (Arita et al., 2014). According to our search, it has not yet been documented for bioaugmentation or biosorption uses. However, *A. parasiticus* isolated from the wastewater of the Rakta pulp factory have been shown to remove 86% of dye contaminants (El-Rahim and Moawad, 2003). Furthermore, Medaura et al. (2021) reported that *A. parasiticus*, combined with other fungal species such as *Penicillium*, *Ulocladium*, *Aspergillus* and *Fusarium*, may be able to biostimulate the high molecular weight polycyclic aromatic hydrocarbons PAHs biodegradation.

*Cladosporium* Link, 1816 is a fungus isolated on various surfaces and comprises species with a wide range of lifestyles (Torres et al., 2017). *Cladosporium cladosporioides* isolated in this study are related to the *Cladosporium* sp. isolated from heavy metal contaminated soils of Turda, Cluy County in Romania. In a previous tolerance experiment, it displayed high resistance to Hg, Pb, Cu, Zn and Cd (Văcar et al., 2021). Similar *C. Cladosporioides* strains recovered from Rudnany in Slovakia were also among the fungal species with a remarkable capacity for mercury removal and volatilization (Šimonovičová et al., 2019). The above findings confirm that the fungi community and capacity for heavy metal resistant varies depending on the site of isolation, local and seasonal climates, soil composition and properties, and isolates metal specificity.

*Geotrichum candidum* Link (1809) is distributed across the world and has been isolated from various places. Examples include sewage sludge (More et al., 2010), tomato (Burlinson et al., 2011), citrus fruit (Talibi et al., 2012), and surface sediments (He et al., 2022). In the present study, *G. candidum* has been detected in soil from the Nkana Slag Dump and Uchi Tailing Dam in Kitwe. Previous studies have identified *G. candidum* among the fungal species capable of metabolizing hydrocarbons, textile dyes and heavy metals contaminants. Therefore, it is classified among the potential species considered as diverse contaminants bioremediators (Goltapeh et al., 2013; Ezekoye et al., 2018). Another study by He et al. (2022) isolated *G. candidum* from surface soil samples from the Futian Mangrove Nature Reserve in Shenzhen, China. The isolates demonstrated good tolerance to Cu^2+^, Zn^2+^, and Ni^2+^. On YM agar, *Geotrichum sp.* CS-67 could tolerate Cu^2+^, Zn^2+^, and Ni^2+^ at maximum concentrations of 350, 900, and 100 mg/L, respectively. The tolerance capacity to Cu, Co, Fe, Mn, Zn, and Pb by our *G. candidum* isolates is far higher than in previous studies because it was isolated from a copper-contaminated environment (Ezzouhri et al., 2009; Muñoz et al., 2012).

In difficult environmental conditions, bioaugmentation is frequently thought to be able to handle bioremediation of the most resistant chemicals (Medaura et al., 2021). This study has tested a fungal consortium that is in charge of more effective biomineralization. Such a concoction may facilitate effective treatment of contaminated soil. Fungi have diverse survival mechanisms in contaminated environments, such as using cell walls to bind metals, intracellular/extracellular enzyme production, intracellular/extracellular metal sequestration, precipitation, suppressed metal influx, enhanced metal efflux, and complexation. Biosorption is among the critical mechanisms fostering tolerance while allowing fungi to survive in harsh environments (Vázquez-Campos et al., 2015; Coelho et al., 2020; Priyadarshini et al., 2021). This makes tailing soils function as metal-rich ecosystems that provide a metal-stressed environment for the evolution of metal-resistant microbiota (Pal et al., 2006).

In order to integrate soil fungi in the bioremediation process of heavy metal contaminated soil and limit pest and disease concerns in phytoremediation, it is necessary to know their phytopathogenicity, in addition to their diverse distribution and dissemination patterns. Various fungi and other microorganisms from all kingdoms attack multiple substrates in the natural ecosystem of soils, and the rate of degradation increases when N, P, and other crucial inorganic elements are present in large quantities (Gadd, 2004). While some plant-pathogenic fungi have a relatively broad host range, the majority have a very restricted range of plant species or even cultivars they may infect. Nonetheless, most plant pathogenic fungi are ubiquitous and endemic in most places, and they are already integrated into most agroecosystems (Termorshuizen, 2017). Therefore, utilization of fungal species in bioremediation programs may not necessarily introduce new hazards into ecosystems. Future studies should evaluate the selected fungi for pathogenicity before further application in large-scale bioremediation programs.

### Fungal heavy metal tolerance capacity

A high level of heavy metal contamination in soil, water, sediment, and overburden can stimulate tolerance and resistance mechanisms of fungi, demonstrating fungi’s potential for utilization in bioremediation. Indigenous microorganisms, particularly fungi obtained from polluted areas, can be trained to enhance their tolerance and biosorption capacity for heavy metals (Wood et al., 2016; Migahed et al., 2017; Oladipo et al., 2018; Coelho et al., 2020).

In this investigation, the presence of highly metal-tolerant strains of *A. transmontanensis, C. cladosporioides*, and *G. candidum* was confirmed at Cu and Co-contaminated sites in Kitwe. Similar studies have reported occurrence of fungi species in other polluted areas with high heavy metal concentrations (Sabra et al., 2012; Talukdar et al., 2020). In particular, Oladipo et al. (2018) found fungal species in soil with high Cd, Cu, Pb, As, and Fe concentrations. Similar findings by Zafar et al. (2007) and (Iskandar et al., 2011; Wong et al., 2018) reported the presence of fungal species in soils and water contaminated with high Cu, Cd, Zn, and As concentrations. Research by Khan et al. (2019b) also confirmed the occurrence of heavy metal tolerant fungal strains in discharged effluent sites of two industries contaminated with heavy metals.

The ability of filamentous fungi to form mycelial structures that can span over a wide range surfaces facilitates fungi to access and remove heavy metal pollutants from the soil. In addition, fungi use both extracellular and intracellular mechanisms to tolerate heavy metals and prevent them from disrupting metal-sensitive cellular targets (Robinson et al., 2021). In most cases, extracellular mechanisms are used to prevent metal absorption, whereas intracellular mechanisms aims to minimize the amount of metal in the cytosol. In extracellular processes, the fungal cell wall extracts various organic compounds that do not belong in the cell wall matrix in order to chelate metal ions (Bellion et al., 2006). Because fungi have negatively charged cell surfaces from the presence of numerous anionic components like glucan, and chitin, metal cations are bound to the cell wall during biosorption (Dhankhar and Hooda, 2011). Metal transport proteins may contribute to metal tolerance in intracellular processes by preventing toxic metal ions either from entering the cytosol, the cell itself, or by facilitating metal sequestration into vacuole compartments (Anahid et al., 2011). The mechanisms of heavy metal tolerance and biosorption of filamentous fungi have been discussed in detail in previous studies and reviews by Bellion et al. (2006); Harms et al. (2011); Dusengemungu et al. (2020).

### Dual interactions In petri dishes

Fungal interactions are frequently the primary drivers of change in fungal populations and the outcomes of heavy metal absorption (Boddy, 2016). In this study, the fungi isolates *G. candidum*, *A. transmontanensi*s had fast growth rates while *C. cladosporioides* had slow growth rate in the PDA medium. Similar interaction patterns were observed during the *in vitro* co-incubation of the three fungal isolates evaluated. *C. cladosporioides* vs. *G. candidum*, *A. transmontanensi*s vs. *G. candidum*, and *C. cladosporioides* vs. *A. transmontanensis* displayed mutual intermingling. In each case, both isolates evolved and progressed into each other’s domains through mutual intermingling. The unrestricted proliferation of both fungal isolates’ mycelia on PDA media proved that they were interacting with one another (Figure 3). However, after the maximum number of incubation days, *G. candidum* showed intense competition for space against both *C. cladosporioides* and *A. transmontanensis* due to its rapid mycelial growth and high capacity in utilizing nutrients in the media*. A. transmontanensis* expanded over and around *C. cladosporioides* due to its spores growth pattern, while the growth of *C. cladosporioides* remained intact. The same findings were reported by Kausar et al. (2010). Complete mutual intermingling was observed between 16% of fungal isolates tested, and partial mutual intermingling and inhibited growth were also observed. In another study, Stahl and Christensen (1992) reported 47% mutual intermingling contacts among soil micro-fungal communities. Although, the interactions between dominant fungal species and other microorganisms might have a particular influence on the bioaugmentation potential of the fungal community in contaminated soil, further research is needed to evaluate the effect of interactions between fungal biodiversity and other microorganisms in heavy metal contaminated environments to develop novel, environmentally friendly bioremediation techniques.

### Heavy metal bioremoval by fungi

Diverse fungal isolates were employed for the mycoremediation. The formulations were based on three primary characteristics. First, the fact that the fungi are indigenous to the polluted soil may give higher effectiveness during the modification of the chemical form of the metals, compared to the use of non-indigenous fungi. Second, the isolation of fungi from the soil suggests the existence of an active metabolism. Third, the resistance of the isolated fungi to heavy metal pollution implies the possibility of biomineralization, bioaccumulation and biosorption ability of the fungi (Emenike et al., 2016; Wood et al., 2016). Three strains of fungi resistant to heavy metals were mixed with equal ratio and inoculated into the polluted soils. Our hypothesis is that they got dominant position over each other depending on their tolerance capacity, spores dispersion, and filamentous growth style. Therefore, further research may focus on the effect of initial ratio among various heavy metal resistant fungi on the removal efficiency and fungal community in the soils.

Our results correlate with the findings by Hassan et al. (2019), whereby the pH soil reaction of the landfill leachate of the polluted soil had a pH that varied between 6.4 and 7.9 on the initial day of the treatment. However, our results differed at the end of the treatment. The pH of the treated soil dropped and varied between 5.4 and 6.9. It was noticed that there was a pH variation between the initial day of treatment and the 90^th^ day of treatment, probably due to the activity of both fungi and other indigenous microorganisms inhabiting the treated soil. The observed steady acidification of the amended contaminated soil was also associated with the action of fungal species due to their capacity to release acidic organic compounds, which reduce the treated soil’s overall pH (Anand et al., 2006; Rousk et al., 2010; Wang et al., 2015).

Copper removal increased with increasing incubation duration (Figure 5A). The application of *A. transmontanensis, Cladosporium*, and *G. candidum* to treat the soil contaminated with high copper concentration showed high efficacy in reducing copper concentration. In some samples, the final concentration was below the international minimum acceptable level in agricultural soil of 38.90 ppm (Staniland et al., 2010). Following amending the soil from the Black Mountain with fungi, the residual copper concentration after 90 days of treatment varied between 0 and 50 ppm. The only exception was observed in treated overburdened soil, where the copper concentration was reduced from 256 ppm to 108 ppm after 90 days of treatment. The low efficacy was attributed to the coarse texture of the overburdened soil treated (Figure 5A). Numerous processes essential to soil functioning, such as density, porosity, infiltration, drainage, aeration, water-holding capacity, erosivity, and biodegradation, are influenced by soil texture (Arriaga et al., 2017; Santos et al., 2019; Prescott et al., 2020).

In Nkana Slag Dump soils, the bioaugmentation of copper-contaminated soil with fungal organisms has shown a reasonable difference between the first and last days of treatment in copper content. This could be explained by the possibility that the treated soil already had some fungi and our treatment with consistent watering increased metabolic activities. However, the data from TD 26, between the first day and last day of treatment, show a slow rate in reducing the concentration of Cu, probably due to exhaustion of nutrients and inhibition by some toxic metabolites. Remarkable Cu biosorption and bioaccumulation by filamentous fungi have been reported previously (Tsekova et al., 2007; Tuzen et al., 2007; Jayaraman and Arumugam, 2014; Wong et al., 2018). The Cu bioremoval includes ion exchange, complexation, intracellular compartmentalization and sequestration, precipitation, and transformation (Malik, 2004; Dusengemungu et al., 2021).

Figure 5B shows the removal of Co by fungi. The concentration of Co decreased from day 1 to day 90. The Co concentration was reduced to almost zero in the amended soil with the highly tolerant fungi. This was attributed to the low level of Co and the highest resistance developed by the fungi against this metal compared to other metals. A progressive and uniform decrease in heavy metal content indicates fungi colonisation efficiency, especially in highly polluted soil. Hence, the Co decontamination of soil was successful. In some samples, Co was not detected, which indicates that the fungus used has a high potential for Co biosorption from low concentrations, even in the presence of high concentrations of Cu and Fe in the soil. Previous studies have reported various fungal biomass (*Aspergillus niger, Aspergillus flavus, Penicillium citrinum, Mucor recemus, Rhizopus Chinensis, Trichoderma viridae, Neurospora crassa*) can be used to decontaminate ^60^Co polluted solutions. Among the fungal isolates tested using dry biomass, the authors demonstrated a high potential for biosorption of Co between 8–500 ng/g of soil, which aligns with our findings (Rashmi et al., 2004; Acosta-Rodríguez et al., 2018). Other studies have reported exceptional capacity of three fungal isolates: *Paecilomyces* sp., *Penicillium* sp., and *Aspergillus transmontanensis Niger* in the biosorption of Co (II) from the aqueous solution, which was 100, 100, and 96.4%, respectively, after 7 days of incubation of fungal biomasses in the aqueous solution. The high efficiency was attributed to the fact that the fungi species were incubated in the aqueous solution, which allowed high contact of fungi biomass with Co.

The bioremoval of Fe in the contaminated soil varied between 33.7 and 91.5%. Previous studies by Hassan et al. (2019) reported a bioremoval efficiency of Fe by fungi in the treated soil of 35%. Free Fe ions, low-affinity Fe chelates, siderophore-Fe chelates, transferrin, heme, and hemoglobin, are a few of the many types of iron that fungi may absorb with remarkable efficiency (Philpott, 2006; Ahemad and Kibret, 2014). Therefore, our results confirm a high removal of Fe (Figure 5C). Fungal growth requires the presence of iron, which fungi can obtain and store in their biomass to enable survival in iron-limited environments. To make it easier for them to acquire insoluble iron, Fungi have evolved different strategies. Creating specific chelating chemicals and low molecular weight organic acids are two of the most pertinent startegies (Comensoli et al., 2017).

In our study, the bioremoval of Mn by fungi varied between 58.5 and 100% (Figure 5D). Previous studies have reported that various fungal species from the phyla of Ascomycota and Basidiomycota can oxidize Mn (Amorim et al., 2018). More studies have found that Arbuscular Mycorrhizal Fungi (AMF) can facilitate cleaning heavy metal-polluted soil due to their capacity for soil aggregation. Indigenous AMF (*Scutellospora reticulata* and *Glomus pansihalos)* have been tested for their potential to stimulate phytoremediation of soils contaminated with Al and Mn, and it was found that *S. reticulata* and *G. pansihalos* significantly minimize the content of Al and Mn in the soils (Alori and Fawole, 2012; Ullah et al., 2019). Although the concentration of Mn from the waste soil samples did not exceed the international allowed concentration of manganese in agricultural soil, our fungal isolates had Mn remarkable tolerance and high bioremediation capacity to be usable for treatment of soil, mining effluent, discharge of manganese, and drainage of mining water from mine quarries (Das et al., 2015; Mohanty et al., 2017).

Figure 5E shows removal of Zn by fungi. The concentration of Zinc in the mine waste soil was below 2.5 ppm, while the maximum tolerance capacity of all the fungal isolates was above 5,000 ppm, which explains why after 90 days, there was no detectable Zinc in our soil. According to Siddiquee et al. (2013), the maximum tolerance and uptake capacity of Zn^2+^ by *Trichoderma Virens* was above 1,200 mg/L Compared with our results, it can be concluded that fungal isolates can grow under a high concentration of heavy metals (Siddiquee et al., 2013). Zinc uptake by indigenous mycorrhizal fungi, *Funneliformis geosporum* (Nicol. & Gerd.) Walker & Schuler have been demonstrated to degrade Zinc contaminants in Zn-stressed plants (Abu-Elsaoud et al., 2017). Manganese and Zinc are chemically similar because they are strong Lewis acids; therefore, they serve a common chemical function as an electrophilic prosthetic group in various enzymes. However, Zn^2+^ uses tetrahedral for coordination chemistry, while Mn^2+^ favours octahedral coordination (Gadd, 1993; Griffin et al., 1995). Few studies have investigated fungi’s Zn^2+^ and Mn^2+^ uptake mechanisms in a metal-contaminated soil.

Figure 5F shows removal of Pb by fungi. The overall concentration of Pb in the mine waste soil varied between 0.15 and 8.67 ppm. A recent study by Khan et al. (2019a) showed that in SDB medium, *A. flavus* from the contaminated soil samples from Hattar Industrial Estate, Pakistan had greater Pb and Hg removal efficiencies (99.20 and 99.30%, respectively). In addition, *A. niger* and *A. terreus* from the same soil samples also demonstrated greater efficacy for Hg removal in YPG medium (96 and 95.50%, respectively). The above results correlate with the findings in this study. More studies have also shown that a diverse range of fungi has exceptional Pb tolerance capacity (Dursun et al., 2003; Adeyemi and Gadd, 2005; Oladipo et al., 2018).

The potential of these Ascomycota fungi to remove heavy metals can be further exploited for bioremediation. Overall, the efficient removal of heavy metals depends on several metal properties, such as electronegativity, atomic mass, and ionic or atomic radius (Gola et al., 2016). Studies have indicated that fungi’s diverse mechanisms of active metal uptake, accumulation, biosorption, cellular precipitation, and valence transformation are activated depending on the type and nature of the metal (Iram et al., 2015; Dusengemungu et al., 2021). In addition, considering that Cu, Fe, Mn, Zn, Co are essential elements for fungal growth, their removal is exceptionally high.

In the present study, a viable technique for decontamination of a heavy metal-polluted environment was shown. This consists in bioaugmentation using a blend of three autochthonous filamentous fungi. It is hypothesized that blending indigenous filamentous fungi for heavy metal bioremediation would generate much better results than using single strains. The present technology should be combined with other decontamination technologies, such as phytoremediation and organic amendments, to enhance autochthonous colonization, thereby stimulating the reclamation process.

## Conclusion

The role of filamentous fungi in post-mining landscape restoration is still unclear. Filamentous fungi are primarily not implicated in the process except for their vital involvement in forming soil structure. Significant research has shown fungi’s capacity in the presence of environmental hazards. Fungal species are highly diverse, and they have been found to survive in extreme environments such as heavy metal-polluted areas. Our study isolated three filamentous fungi species endemic in heavy metal-contaminated sites. These were *Aspergillus transmontanensis, Cladosporium cladosporioides,* and *Geotrichum candidum* spp. These fungi were shown to not only thrive in such environments but also to be able to significantly reduce the concentrations of six heavy elements from the soil, namely, Cu, Co, Zn, Fe, Mn and Pb, thus providing more evidence for the importance of fungi species during remediation of heavy metal-contaminated soil. However, their inoculation for a more comprehensive application would require more research to elucidate if they are genetically stable and do not produce toxic metabolites. Further research is necessary to study the mechanism by which the fungi select, degrade, and absorb various metals and their influence on other microbiota. Alternatively, our sampling attempt has covered only a tiny portion of the polluted area, and a small fraction of the fungi diversity may have been isolated. Therefore, further studies are still needed to elucidate a broader range of fungi diversity in post-mining areas.

## Data availability statement

The datasets presented in this study can be found in online repositories. The names of the repository/repositories and accession number(s) can be found at: https://www.ncbi.nlm.nih.gov/genbank/, OP320880, OP320881, OP320882, OP320883, OP320885, OP355458.

## Author contributions

LD designed the proposal, collected samples from the field, and was involved in data analysis, wrote, and prepared the tables and figures for the first draft of this manuscript. GS, BM and CG reviewed the proposal, contributed to critical inputs and edited the last draft. All authors listed have made a substantial, direct, and intellectual contribution to the work and approved it for publication.

## Funding

This work has received funding and support from the Copperbelt University, Africa Centre of Excellence for Sustainable Mining (CBU ACESM).

## Conflict of interest

The authors declare that the research was conducted in the absence of any commercial or financial relationships that could be construed as a potential conflict of interest.

## Publisher’s note

All claims expressed in this article are solely those of the authors and do not necessarily represent those of their affiliated organizations, or those of the publisher, the editors and the reviewers. Any product that may be evaluated in this article, or claim that may be made by its manufacturer, is not guaranteed or endorsed by the publisher.

## Footnotes

^1^ https://mafft.cbrc.jp/alignment/server/index.html

^2^ https://www.atgc-montpellier.fr/phyml/

## References

[ref1] Abu-Elsaoud A. M. Nafady N. A. Abdel-Azeem A. M. (2017). Arbuscular mycorrhizal strategy for zinc mycoremediation and diminished translocation to shoots and grains in wheat. PLoS One 12, 1–21. doi: 10.1371/journal.pone.0188220 PMC5690681 29145471

[ref2] Acosta-Rodríguez I. Cárdenas-González J. F. Rodríguez Pérez A. S. Oviedo J. T. Martínez-Juárez V. M. (2018). Bioremoval of different heavy metals by the resistant fungal strain aspergillus Niger. Bioinorg. Chem. Appl. 2018, 1–7. doi: 10.1155/2018/3457196 PMC6236671 30515192

[ref3] Adeyemi A. O. Gadd G. M. (2005). Fungal degradation of calcium-, lead- and silicon-bearing minerals. Biometals 18, 269–281. doi: 10.1007/s10534-005-1539-2 15984571

[ref4] Ahemad M. Kibret M. (2014). Mechanisms and applications of plant growth promoting rhizobacteria: current perspective. J. King Saud Univ. Sci. 26, 1–20. doi: 10.1016/j.jksus.2013.05.001

[ref5] Ahmad E. Zaidi A. Khan M. S. Oves M. (2012). “Heavy metal toxicity to symbiotic nitrogen-fixing microorganism and host legumes,” in Toxicity of heavy metals to legumes and bioremediation. eds. Zaidi, A. Wani, P. A. Khan, M. Saghir (Vienna: Springer), 29–44.

[ref6] Albert K. M. (2015). Role of revegetation in restoring fertility of degraded mined soils in Ghana: a review. Int. J. Biodivers. Conserv. 7, 57–80. doi: 10.5897/ijbc2014.0775

[ref7] Alori E. Fawole O. (2012). Phytoremediation of soils contaminated with aluminium and manganese by two arbuscular mycorrhizal fungi. J. Agric. Sci. 4, 246–252. doi: 10.5539/jas.v4n8p246

[ref8] Amorim S. S. Ruas F. A. D. Barboza N. R. De Oliveira Neves V. G. Leão V. A. Guerra-Sá R. (2018). Manganese (Mn2+) tolerance and biosorption by Meyerozyma guilliermondii and Meyerozyma caribbica strains. J. Environ. Chem. Eng. 6, 4538–4545. doi: 10.1016/j.jece.2018.06.061

[ref9] Anahid S. Yaghmaei S. Ghobadinejad Z. (2011). Heavy metal tolerance of fungi. Sci. Iran. 18, 502–508. doi: 10.1016/j.scient.2011.05.015

[ref10] Anand P. Isar J. Saran S. Saxena R. K. (2006). Bioaccumulation of copper by Trichoderma viride. Bioresour. Technol. 97, 1018–1025. doi: 10.1016/j.biortech.2005.04.046 16324839

[ref11] Arita C. Calado T. Venâncio A. Lima N. Rodrigues P. (2014). Description of a strain from an atypical population of *Aspergillus parasiticus* that produces aflatoxins B only, and the impact of temperature on fungal growth and mycotoxin production. Eur. J. Plant Pathol. 139, 655–661. doi: 10.1007/s10658-014-0438-1

[ref12] Arriaga F. J. Guzman J. Lowery B. (2017). Conventional agricultural production systems and soil functions. Soil Heal. Intensif. Agroecosystems, 109–125. doi: 10.1016/B978-0-12-805317-1.00005-1

[ref13] Bellion M. Courbot M. Jacob C. Blaudez D. Chalot M. (2006). Extracellular and cellular mechanisms sustaining metal tolerance in ectomycorrhizal fungi. FEMS Microbiol. Lett. 254, 173–181. doi: 10.1111/j.1574-6968.2005.00044.x 16445743

[ref14] Bhandari G. Bhatt P. (2021). “Concepts and application of plant–microbe interaction in remediation of heavy metals,” in Microbes and Signaling biomolecules against plant stress. eds. Sharma, Anita (Singapore: Springer), 55–77.

[ref15] Boddy L. (2016). Interactions between fungi and other microbes. Third Edit. Elsevier Ltd.

[ref16] Bosco F. Mollea C. (2019). “Mycoremediation in Soil,” Environmental Chemistry and Recent Pollution Control Approaches (IntechOpen).

[ref17] Burford E. P. Fomina M. Gadd G. M. (2003). Fungal involvement in bioweathering and biotransformation of rocks and minerals. Mineral. Mag. 67, 1127–1155. doi: 10.1180/0026461036760154

[ref18] Burlinson P. Deveau A. Barret M. Tarkka M. Sarniguet A. (2011). Bacterial-fungal interactions: Hyphens between agricultural, clinical, environmental, and food microbiologists. Microbiol. Mol. Biol. Rev. 75, 583–609. doi: 10.1128/MMBR.00020-11 22126995 PMC3232736

[ref19] Chakravarty R. Banerjee P. C. (2012). Mechanism of cadmium binding on the cell wall of an acidophilic bacterium. Bioresour. Technol. 108, 176–183. doi: 10.1016/j.biortech.2011.12.100 22261660

[ref20] Chileshe M. N. Syampungani S. Festin E. S. Tigabu M. Daneshvar A. Odén P. C. (2019). Physico-chemical characteristics and heavy metal concentrations of copper mine wastes in Zambia: implications for pollution risk and restoration. J. For. Res. 13, 1283–1293. doi: 10.1007/s11676-019-00921-0

[ref21] Coelho E. Reis T. A. Cotrim M. Mullan T. K. Corrêa B. (2020). Resistant fungi isolated from contaminated uranium mine in Brazil shows a high capacity to uptake uranium from water. Chemosphere 248:126068. doi: 10.1016/j.chemosphere.2020.126068 32045976

[ref22] Comensoli L. Bindschedler S. Junier P. Joseph E. (2017). Iron and fungal physiology: a review of biotechnological opportunities. Adv. Appl. Microbiol. 98, 31–60. doi: 10.1016/bs.aambs.2016.11.001 28189154

[ref23] Cook J. M. Gardner M. J. Griffiths A. H. Jessep M. A. Ravenscroft J. E. Yates R. (1997). The comparability of sample digestion techniques for the determination of metals in sediments. Mar. Pollut. Bull. 34, 637–644.

[ref24] Da Opaluwa O. Aremu M. O. Ogbo L. O. Abiola K. A. Odiba I. E. Abubakar M. M.. (2012). Heavy metal concentrations in soils, plant leaves and crops grown around dump sites in Lafia Metropolis, Nasarawa state, Nigeria. Adv. Appl. Sci. Res. 3, 780–784.

[ref25] Das S. K. Das A. R. Guha A. K. (2007). A study on the adsorption mechanism of mercury on aspergillus versicolor biomass. Environ. Sci. Technol. 41, 8281–8287. doi: 10.1021/es070814g 18200852

[ref26] Das A. P. Ghosh S. Mohanty S. Sukla L. B. (2015). “Advances in Manganese Pollution and Its Bioremediation,” in Soil biology series, 313–328.

[ref27] Deshmukh R. Khardenavis A. A. Purohit H. J. (2016). Diverse metabolic capacities of fungi for bioremediation. Indian J. Microbiol. 56, 247–264. doi: 10.1007/s12088-016-0584-6 27407289 PMC4920763

[ref28] Dhankhar R. Hooda A. (2011). Fungal biosorption – an alternative to meet the challenges of heavy metal pollution in aqueous solutions. Environ. Technol. 3330, 467–491. doi: 10.1080/09593330.2011.572922 21877528

[ref29] Dursun A. Y. Uslu G. Cuci Y. Aksu Z. (2003). Bioaccumulation of copper(II), lead(II) and chromium(VI) by growing aspergillus Niger. Process Biochem. 38, 1647–1651. doi: 10.1016/S0032-9592(02)00075-4

[ref30] Dusengemungu L. Kasali G. Gwanama C. Mubemba B. (2021). Overview of fungal bioleaching of metals. Environ. Adv. 5:100083. doi: 10.1016/j.envadv.2021.100083

[ref31] Dusengemungu L. Kasali G. Gwanama C. Ouma K. O. (2020). Recent advances in biosorption of copper and cobalt by filamentous fungi. Front. Microbiol. 11, 1–16. doi: 10.3389/fmicb.2020.582016 33408701 PMC7779407

[ref32] Dusengemungu L. Mubemba B. Gwanama C. (2022). Evaluation of heavy metal contamination in copper mine tailing soils of Kitwe and Mufulira, Zambia, for reclamation prospects. Sci. Rep. 12, 1–16. doi: 10.1038/s41598-022-15458-2 35787645 PMC9253116

[ref33] El-Rahim W. M. A. Moawad H. (2003). Enhancing bioremoval of textile dyes by eight fungal strains from media supplemented with gelatine wastes and sucrose. J. Basic Microbiol. 43, 367–375. doi: 10.1002/jobm.200310267 12964179

[ref34] Emenike C. U. Agamuthu P. Fauziah S. H. (2017). Sustainable remediation of heavy metal polluted soil: a biotechnical interaction with selected bacteria species. J. Geochem. Explor. 182, 275–278. doi: 10.1016/j.gexplo.2016.10.002

[ref35] Esshaimi M. El Gharmali A. Berkhis F. Valiente M. Mandi L. (2017). Speciation of heavy metals in the soil and the mining residues, in the Zinclead Sidi Bou Othmane abandoned mine in Marrakech area. Linnaeus Eco-Tech., 975–985. doi: 10.15626/eco-tech.2010.102

[ref01] Ettler V. Mihaljevič M. Kříbek B. Majer V. Šebek O. (2011). Tracing the spatial distribution and mobility of metal/metalloid contaminants in Oxisols in the vicinity of the Nkana copper smelter, Copperbelt province, Zambia. Geoderma 164, 73–84. doi: 10.1016/j.geoderma.2011.05.014

[ref36] Ezekoye C. C. Chikere C. B. Okpokwasili G. C. (2018). Fungal diversity associated with crude oil-impacted soil undergoing in-situ bioremediation. Sustain. Chem. Pharm. 10, 148–152. doi: 10.1016/j.scp.2018.11.003

[ref37] Ezzouhri L. Castro E. Moya M. Espinola F. Lairini K. (2009). Heavy metal tolerance of filamentous fungi isolated from polluted sites in Tangier, Morocco. Afr. J. Microbiol. Res. 3, 35–48. doi: 10.5897/AJMR.9000354

[ref38] Emenike C. U. Agamuthu P. Fauziah S. H. (2016). Blending Bacillus sp., Lysinibacillus sp. and Rhodococcus sp. for optimal reduction of heavy metals in leachate contaminated soil. Environ. Earth Sci. 75:26 doi: 10.1007/s12665-015-4805-9

[ref39] Fawzy E. M. Abdel Motaal F. F. El Zayat S. A. (2017). Biosorption of heavy metals onto different eco-friendly substrates. J. Bioremed. Biodegr. 08. doi: 10.4172/2155-6199.1000394

[ref40] Festin E. S. Tigabu M. Chileshe M. N. Syampungani S. Odén P. C. (2019). Progresses in restoration of post-mining landscape in Africa. J. For. Res. 30, 381–396. doi: 10.1007/s11676-018-0621-x

[ref41] Gadd G. M. (1993). Tansley review no. 47. Interactions of fungi with toxic metals. New Phytol. 124, 25–60.

[ref42] Gadd G. M. (2004). Mycotransformation of organic and inorganic substrates. Mycologist 18, 60–70. doi: 10.1017/S0269915X04002022

[ref43] Gadd G. M. Bahri-Esfahani J. Li Q. Rhee Y. J. Wei Z. Fomina M.. (2014). Oxalate production by fungi: significance in geomycology, biodeterioration and bioremediation. Fungal Biol. Rev. 28, 36–55. doi: 10.1016/j.fbr.2014.05.001

[ref44] Gathuru G. (2011). The performance of selected tree species in the rehabilitation of a limestone quarry at east African Portland cement company land, Athi River, Kenya.

[ref45] Gente S. Sohier D. Coton E. Duhamel C. Gueguen M. (2006). Identification of Geotrichum candidum at the species and strain level: proposal for a standardized protocol. J. Ind. Microbiol. Biotechnol. 33, 1019–1031. doi: 10.1007/s10295-006-0130-3 16855820

[ref46] Gherbawy Y. Voigt K. (2010). Molecular identification of fungi.

[ref47] Gola D. Dey P. Bhattacharya A. Mishra A. Malik A. Namburath M.. (2016). Multiple heavy metal removal using an entomopathogenic fungi Beauveria bassiana. Bioresour. Technol. 218, 388–396. doi: 10.1016/j.biortech.2016.06.096 27387415

[ref48] Goltapeh E. M. Danesh Y. R. Varma A. (2013). Fungi as Bioremediators. eds. E. M. Goltapeh, Y. R. Danesh, and A. Varma (Berlin, Heidelberg: Springer).

[ref49] Griffin D. H. Winkelmann G. Winge D. R. (1995). Metal Ions in Fungi.

[ref50] Guindon S. Lethiec F. Duroux P. Gascuel O. (2005). PHYML online--a web server for fast maximum likelihood-based phylogenetic inference. Nucleic Acids Res. 33, W557–W559. doi: 10.1093/nar/gki352 15980534 PMC1160113

[ref51] Gupta S. Wali A. Gupta M. Annepu S. K. (2017). “Fungi: an effective tool for bioremediation,” in Plant-microbe interactions in Agro-ecological perspectives. eds. Dr. Dhananjaya Pratap Singh, H. B. Singh, and R. Prabha (Singapore: Springer Singapore), 593–606.

[ref52] Halfeld-Vieira B. A. Terao D. Nechet K. L. (2015). First report of Geotrichum candidum causing sour-rot of melon in Brazil. Plant Dis. 95, 10–11. doi: 10.1094/PDIS-11-19-2484-PDN

[ref53] Hansda A. Kumar V. Anshumali (2016). A comparative review towards potential of microbial cells for heavy metal removal with emphasis on biosorption and bioaccumulation. World J. Microbiol. Biotechnol. 32:170. doi: 10.1007/s11274-016-2117-1 27565780

[ref54] Harms H. Schlosser D. Wick L. Y. (2011). Untapped potential: exploiting fungi in bioremediation of hazardous chemicals. Nat. Rev. Microbiol. 9, 177–192. doi: 10.1038/nrmicro2519 21297669

[ref55] Hassan A. Pariatamby A. Ahmed A. Auta H. S. Hamid F. S. (2019). Enhanced bioremediation of heavy metal contaminated landfill soil using filamentous fungi consortia: a demonstration of bioaugmentation potential. Water Air Soil Pollut. 230:215. doi: 10.1007/s11270-019-4227-5

[ref56] Hassan A. Pariatamby A. Ossai I. C. Hamid F. S. (2020). Bioaugmentation assisted mycoremediation of heavy metal and/metalloid landfill contaminated soil using consortia of filamentous fungi. Biochem. Eng. J. 157:107550. doi: 10.1016/j.bej.2020.107550

[ref57] He M. Xu Y. Qiao Y. Zhang Z. Liang J. Peng Y.. (2022). A novel yeast strain Geotrichum sp. CS-67 capable of accumulating heavy metal ions. Ecotoxicol. Environ. Saf. 236:113497. doi: 10.1016/j.ecoenv.2022.113497 35405529

[ref58] Iram S. Shabbir R. Zafar H. Javaid M. (2015). Biosorption and bioaccumulation of copper and Lead by heavy metal-resistant fungal isolates. Arab. J. Sci. Eng. 40, 1867–1873. doi: 10.1007/s13369-015-1702-1

[ref59] Iskandar N. L. Zainudin N. A. I. M. Tan S. G. (2011). Tolerance and biosorption of copper (cu) and lead (pb) by filamentous fungi isolated from a freshwater ecosystem. J. Environ. Sci. 23, 824–830. doi: 10.1016/S1001-0742(10)60475-5 21790056

[ref60] Jayaraman M. Arumugam R. (2014). Biosorption of copper (II) by aspergillus flavus. Int. J. Sci. Res. 3, 335–340.

[ref61] Joshi P. K. Swarup A. Maheshwari S. Kumar R. Singh N. (2011). Bioremediation of heavy metals in liquid media through fungi isolated from contaminated sources. Indian J. Microbiol. 51, 482–487. doi: 10.1007/s12088-011-0110-9 23024411 PMC3209935

[ref62] Kapungwe E. M. (2013). Heavy metal contaminated water, soils and crops in peri urban wastewater irrigation farming in Mufulira and Kafue towns in Zambia. J. Geogr. Geol. 5. doi: 10.5539/jgg.v5n2p55

[ref63] Katoh K. Rozewicki J. Yamada K. D. (2019). MAFFT online service: multiple sequence alignment, interactive sequence choice and visualization. Brief. Bioinform. 20, 1160–1166. doi: 10.1093/bib/bbx108 28968734 PMC6781576

[ref64] Kausar H. Sariah M. Mohd Saud H. Zahangir Alam M. Razi Ismail M. (2010). Development of compatible lignocellulolytic fungal consortium for rapid composting of rice straw. Int. Biodeterior. Biodegrad. 64, 594–600. doi: 10.1016/j.ibiod.2010.06.012

[ref65] Khan I. Aftab M. Shakir S. U. Ali M. Qayyum S. Rehman M. U.. (2019a). Mycoremediation of heavy metal (cd and Cr)–polluted soil through indigenous metallotolerant fungal isolates. Environ. Monit. Assess. 191:585. doi: 10.1007/s10661-019-7769-5 31440913

[ref66] Khan I. Ali M. Aftab M. Shakir S. U. Qayyum S. Haleem K. S.. (2019b). Mycoremediation: a treatment for heavy metal-polluted soil using indigenous metallotolerant fungi. Environ. Monit. Assess. 191:622. doi: 10.1007/s10661-019-7781-9 31494726

[ref67] Khatri N. Tyagi S. (2015). Influences of natural and anthropogenic factors on surface and groundwater quality in rural and urban areas. Front. Life Sci. 8, 23–39. doi: 10.1080/21553769.2014.933716

[ref68] Leck A. (1999). Preparation of lactophenol cotton blue slide mounts. Community Eye Heal. 12, 30–24. PMC1706009 17491984

[ref69] Lee S. Yamamoto N. (2015). Accuracy of the high-throughput amplicon sequencing to identify species within the genus Aspergillus. Fungal Biol. 119, 1311–1321. doi: 10.1016/j.funbio.2015.10.006 26615752

[ref70] Lefort V. Longueville J. E. Gascuel O. (2017). SMS: smart model selection in PhyML. Mol. Biol. Evol. 34, 2422–2424. doi: 10.1093/MOLBEV/MSX149 28472384 PMC5850602

[ref71] Lestan D. Lestan M. Chapelle J. A. Lamar R. T. (1996). Development of fungal Inocula for bioaugmentation of contaminated soils. J. Ind. Microbiol. 16, 286–294. doi: 10.1007/BF01570036 PMC1388875 16535337

[ref72] Letunic I. Bork P. (2021). Interactive tree of life (iTOL) v5: an online tool for phylogenetic tree display and annotation. Nucleic Acids Res. 49, W293–W296. doi: 10.1093/nar/gkab301 33885785 PMC8265157

[ref73] Liang J.-J. Zhang P.-P. Zhang W. Song D. Wei X. Yin X.. (2022). Biological activities and secondary metabolites from *Sophora tonkinensis* and its endophytic fungi. Molecules 27:5562. doi: 10.3390/molecules27175562 36080327 PMC9457587

[ref74] Lobos A. Harwood V. J. Scott K. M. Cunningham J. A. (2020). Tolerance of three fungal species to lithium and cobalt: implications for bioleaching of spent rechargeable Li-ion batteries. J. Appl. Microbiol. 131, 743–755. doi: 10.1111/jam.14947 33251646

[ref75] Malik A. (2004). Metal bioremediation through growing cells. Environ. Int. 30, 261–278. doi: 10.1016/j.envint.2003.08.001 14749114

[ref76] Mancera-López M. E. Esparza-García F. Chávez-Gómez B. Rodríguez-Vázquez R. Saucedo-Castañeda G. Barrera-Cortés J.. (2008). Bioremediation of an aged hydrocarbon-contaminated soil by a combined system of biostimulation-bioaugmentation with filamentous fungi. Int. Biodeterior. Biodegrad. 61, 151–160. doi: 10.1016/j.ibiod.2007.05.012

[ref77] Medaura M. C. Guivernau M. Moreno-Ventas X. Prenafeta-Boldú F. X. Viñas M. (2021). Bioaugmentation of native fungi, an efficient strategy for the bioremediation of an aged industrially polluted soil with heavy hydrocarbons. Front. Microbiol. 12, 1–18. doi: 10.3389/fmicb.2021.626436 PMC8044458 33868189

[ref78] Migahed F. Abdelrazak A. Fawzy G. (2017). Batch and continuous removal of heavy metals from industrial effluents using microbial consortia. Int. J. Environ. Sci. Technol. 14, 1169–1180. doi: 10.1007/s13762-016-1229-3

[ref79] Mohanty S. Ghosh S. Nayak S. Das A. P. (2017). Isolation, identification and screening of manganese solubilizing fungi from low-grade manganese ore deposits. Geomicrobiol J. 34, 309–316. doi: 10.1080/01490451.2016.1189016

[ref80] Moll J. Hoppe B. König S. Wubet T. Buscot F. Krüger D. (2016). Spatial distribution of fungal communities in an arable soil. PLoS One 11:e0148130. doi: 10.1371/journal.pone.0148130 26840453 PMC4740416

[ref81] More T. T. Yan S. Tyagi R. D. Surampalli R. Y. (2010). Potential use of filamentous fungi for wastewater sludge treatment. Bioresour. Technol. 101, 7691–7700. doi: 10.1016/j.biortech.2010.05.033 20542684

[ref82] Moretti A. (2017). Mycotoxigenic Fungi. eds. A. Moretti and A. Susca (New York, NY: Springer).

[ref83] Muñoz A. J. Ruiz E. Abriouel H. Gálvez A. Ezzouhri L. Lairini K.. (2012). Heavy metal tolerance of microorganisms isolated from wastewaters: identification and evaluation of its potential for biosorption. Chem. Eng. J. 210, 325–332. doi: 10.1016/j.cej.2012.09.007

[ref84] Murugesan K. Selvam A. Wong J. W. C. (2014). Flocculation and dewaterability of chemically enhanced primary treatment sludge by bioaugmentation with filamentous fungi. Bioresour. Technol. 168, 198–203. doi: 10.1016/j.biortech.2014.04.063 24878139

[ref85] Oladipo O. G. Awotoye O. O. Olayinka A. Bezuidenhout C. C. Maboeta M. S. (2018). Heavy metal tolerance traits of filamentous fungi isolated from gold and gemstone mining sites. Braz. J. Microbiol. 49, 29–37. doi: 10.1016/j.bjm.2017.06.003 28844883 PMC5790576

[ref86] Pal A. Ghosh S. Paul A. K. (2006). Biosorption of cobalt by fungi from serpentine soil of Andaman. Bioresour. Technol. 97, 1253–1258. doi: 10.1016/j.biortech.2005.01.043 16023340

[ref87] Pehoiu G. Murarescu O. Radulescu C. Dulama I. D. Teodorescu S. Stirbescu R. M.. (2020). Heavy metals accumulation and translocation in native plants grown on tailing dumps and human health risk. Plant Soil 456, 405–424. doi: 10.1016/S0009-2541(00)00422-8

[ref88] Pettersson U. T. Ingri J. (2001). The geochemistry of co and cu in the Kafue River as it drains the Copperbelt mining area, Zambia. Chem. Geol. 177, 399–414. doi: 10.1016/S0009-2541(00)00422-8

[ref89] Philpott C. C. (2006). Iron uptake in fungi: a system for every source. Biochim. Biophys. Acta, Mol. Cell Res. 1763, 636–645. doi: 10.1016/j.bbamcr.2006.05.008 16806534

[ref90] Pourret O. Lange B. Bonhoure J. Colinet G. Decrée S. Mahy G.. (2015). Assessment of soil metal distribution and environmental impact of mining in Katanga (Democratic Republic of Congo). Appl. Geochem. 64, 43–55. doi: 10.1016/j.apgeochem.2015.07.012

[ref91] Prescott C. E. Katzensteiner K. Weston C. (2020). Soils and restoration of forested landscapes. Soils Landsc. Restor., 299–331. doi: 10.1016/B978-0-12-813193-0.00011-4

[ref92] Priyadarshini E. Priyadarshini S. S. Cousins B. G. Pradhan N. (2021). Metal-fungus interaction: review on cellular processes underlying heavy metal detoxification and synthesis of metal nanoparticles. Chemosphere 274:129976. doi: 10.1016/j.chemosphere.2021.129976 33979913

[ref93] R Core Team (2020). R: A language and environment for statistical computing. *R Found. Stat. Comput.* Vienna, Austria. Available at: https://www.r-project.org/. (Accessed August 10, 2022).

[ref94] Rambaut A. Lam T. T. Carvalho L. M. Pybus O. G. (2016). Exploring the temporal structure of heterochronous sequences using TempEst (formerly path-O-gen). Virus Evol. 2, 1–7. doi: 10.1093/ve/vew007 PMC4989882 27774300

[ref95] Rashmi K. Sowjanya T. N. Mohan P. M. Balaji V. Venkateswaran G. (2004). Bioremediation of 60Co from simulated spent decontamination a solutions. Sci. Total Environ. 328, 1–14. doi: 10.1016/j.scitotenv.2004.02.009 15207568

[ref96] Ren W. X. Li P. J. Geng Y. Li X. J. (2009). Biological leaching of heavy metals from a contaminated soil by *Aspergillus niger*. J. Hazard. Mater. 167, 164–169. doi: 10.1016/j.jhazmat.2008.12.104 19232463

[ref97] Robinson J. R. Isikhuemhen O. S. Anike F. N. (2021). Fungal–metal interactions: a review of toxicity and homeostasis. J. Fungi 7:225. doi: 10.3390/jof7030225 PMC8003315 33803838

[ref98] Rousk J. Brookes P. C. Bååth E. (2010). Investigating the mechanisms for the opposing pH relationships of fungal and bacterial growth in soil. Soil Biol. Biochem. 42, 926–934. doi: 10.1016/j.soilbio.2010.02.009

[ref99] RStudio (2016). RStudio: Integrated development environment for R. RStudio, Inc. Boston, MA, USA,.

[ref100] Sabra N. Dubourguier H.-C. Hamieh T. (2012). Fungal leaching of heavy metals from sediments dredged from the Deûle Canal, France. Adv. Chem. Eng. Sci. 02, 1–8. doi: 10.4236/aces.2012.21001

[ref101] Samson R. A. Visagie C. M. Houbraken J. Hong S.-B. Hubka V. Klaassen C. H. W.. (2014). Phylogeny, identification and nomenclature of the genus Aspergillus. Stud. Mycol. 78, 141–173. doi: 10.1016/j.simyco.2014.07.004 25492982 PMC4260807

[ref102] Santos F. Abney R. Barnes M. Bogie N. Ghezzehei T. A. Jin L.. (2019). The role of the physical properties of soil in determining biogeochemical responses to soil warming. Ecosyst. Consequences Soil Warm. Microbes, Veg. Fauna Soil Biogeochem., 209–244. doi: 10.1016/B978-0-12-813493-1.00010-7

[ref103] Schubert K. Groenewald J. Z. Braun U. Dijksterhuis J. Starink M. Hill C. F.. (2007). Biodiversity in the Cladosporium herbarum complex (Davidiellaceae, Capnodiales), with standardisation of methods for Cladosporium taxonomy and diagnostics. Stud. Mycol. 58, 105–156. 18490998 10.3114/sim.2007.58.05 PMC2104742

[ref104] Sey E. Belford E. J. D. (2021). Heavy Metals Tolerance Potential of Fungi Species Isolated from Gold Mine Tailings in Ghana.

[ref105] Siddiquee S. Aishah S. N. Azad S. A. Shafawati S. N. Naher L. (2013). Tolerance and biosorption capacity of Zn2+, Pb2+, Ni3+ and Cu2+ by filamentous fungi (Trichoderma harzianum, T. aureoviride and T. virens). Adv. Biosci. Biotechnol. 4, 570–583. doi: 10.4236/abb.2013.44075

[ref106] Silva Í. S. dos Santos E. Da C. De Menezes C. R. De Faria A. F. Franciscon E.. (2009). Bioremediation of a polyaromatic hydrocarbon contaminated soil by native soil microbiota and bioaugmentation with isolated microbial consortia. Bioresour. Technol. 100, 4669–4675. doi: 10.1016/j.biortech.2009.03.079 19477638

[ref107] Simate G. S. Ndlovu S. Walubita L. F. (2010). The fungal and chemolithotrophic leaching of nickel laterites—challenges and opportunities. Hydrometallurgy 103, 150–157. doi: 10.1016/j.hydromet.2010.03.012

[ref108] Šimonovičová A. Kraková L. Pauditšová E. Pangallo D. (2019). Occurrence and diversity of cultivable autochthonous microscopic fungi in substrates of old environmental loads from mining activities in Slovakia. Ecotoxicol. Environ. Saf. 172, 194–202. doi: 10.1016/j.ecoenv.2019.01.064 30708231

[ref109] Singh A. Roy A. (2021) in Fungal communities for the remediation of environmental pollutants. ed. Yadav A. N. (Cham: Springer International Publishing), 127–165.

[ref110] Singh M. Srivastava P. K. Verma P. C. Kharwar R. N. Singh N. Tripathi R. D. (2015). Soil fungi for mycoremediation of arsenic pollution in agriculture soils. J. Appl. Microbiol. 119, 1278–1290. doi: 10.1111/jam.12948 26348882

[ref111] Stahl P. D. Christensen M. (1992). In vitro mycelial interactions among members of a soil microfungal community. Soil Biol. Biochem. 24, 309–316.

[ref112] Staniland S. Coppock M. Tuffin M. van Zyl L. Roychoudhury A. N. Cowan D. (2010). Cobalt uptake and resistance to trace metals in comamonas testosteroni isolated from a heavy-metal contaminated site in the Zambian Copperbelt. Geomicrobiol J. 27, 656–668. doi: 10.1080/01490450903527994

[ref113] Szada-Borzyszkowska A. Krzyżak J. Rusinowski S. Starzewska-Sikorska A. Ratman-Kłosińska I. Pogrzeba M. (2021). The effect of amendments on Lolium perenne roots arbuscular mycorrhizal fungi colonization when cultivated in contaminated soil. Int. J. Environ. Sci. Technol. 19, 1–12. doi: 10.1007/s13762-021-03783-4

[ref114] Talibi I. Askarne L. Boubaker H. Boudyach E. H. Msanda F. Saadi B.. (2012). Antifungal activity of some Moroccan plants against Geotrichum candidum, the causal agent of postharvest citrus sour rot. Crop Prot. 35, 41–46. doi: 10.1016/j.cropro.2011.12.016

[ref115] Talukdar D. Jasrotia T. Sharma R. Jaglan S. Kumar R. R. Vats R.. (2020). Evaluation of novel indigenous fungal consortium for enhanced bioremediation of heavy metals from contaminated sites. Environ. Technol. Innov. 20:101050. doi: 10.1016/j.eti.2020.101050

[ref116] Termorshuizen A. J. (2017). “Ecology of fungal plant pathogens,” in Fungal Kingdom, (Washington, DC, USA: ASM Press), 387–397.

[ref117] Tobin J. M. White C. Gadd G. M. (1994). Metal accumulation by fungi: applications in environmental biotechnology. J. Ind. Microbiol. 13, 126–130.

[ref118] Torres D. E. Rojas-Martínez R. I. Zavaleta-Mejía E. Guevara-Fefer P. Márquez-Guzmán G. J. Pérez-Martínez C. (2017). Cladosporium cladosporioides and Cladosporium pseudocladosporioides as potential new fungal antagonists of Puccinia horiana Henn., the causal agent of chrysanthemum white rust. PLoS One 12, 1–16. doi: 10.1371/journal.pone.0170782 PMC5283677 28141830

[ref119] Tsekova K. Ianis M. Ganeva S. (2007). Biosorption of binary mixtures of copper and cobalt by penicillium cyclopium biomass. Comptes Rendus L’Academie Bulg. des Sci. 60, 63–70. doi: 10.1515/znc-2007-3-417 17542494

[ref120] Tutu H. McCarthy T. S. Cukrowska E. (2008). The chemical characteristics of acid mine drainage with particular reference to sources, distribution and remediation: the Witwatersrand Basin, South Africa as a case study. Appl. Geochem. 23, 3666–3684. doi: 10.1016/j.apgeochem.2008.09.002

[ref121] Tuzen M. Uluozlu O. D. Usta C. Soylak M. (2007). Biosorption of copper(II), lead(II), iron(III) and cobalt(II) on Bacillus sphaericus-loaded Diaion SP-850 resin. Anal. Chim. Acta 581, 241–246. doi: 10.1016/j.aca.2006.08.040 17386450

[ref122] Ullah R. Hadi F. Ahmad S. (2019). Phytoremediation of Lead and chromium contaminated soil improves with the endogenous phenolics and proline production in *Parthenium*, *Cannabis*, *Euphorbia*, and *Rumex* species. Water Air Soil Pollut. 230:40. doi: 10.1007/s11270-019-4089-x

[ref123] Văcar C. L. Covaci E. Chakraborty S. Li B. Weindorf D. C. Frențiu T.. (2021). Heavy metal-resistant filamentous fungi as potential mercury bioremediators. J. Fungi 7:386. doi: 10.3390/jof7050386 PMC8156478 34069296

[ref124] Vázquez-Campos X. Kinsela A. S. Collins R. N. Neilan B. A. Aoyagi N. Waite T. D. (2015). Uranium binding mechanisms of the acid-tolerant fungus Coniochaeta fodinicola. Environ. Sci. Technol. 49, 8487–8496. 26106944 10.1021/acs.est.5b01342

[ref125] Velkova Z. Kirova G. Stoytcheva M. Kostadinova S. Todorova K. Gochev V. (2018). Immobilized microbial biosorbents for heavy metals removal. Eng. Life Sci. 18, 871–881. doi: 10.1002/elsc.201800017 32624881 PMC6999454

[ref126] Visagie C. M. Houbraken J. (2020). Updating the taxonomy of aspergillus in South Africa. Stud. Mycol. 95, 253–292. doi: 10.1016/j.simyco.2020.02.003 32855741 PMC7426233

[ref127] Vítková M. Ettler V. Johan Z. Kříbek B. Šebek O. Mihaljevič M. (2010). Primary and secondary phases in copper-cobalt smelting slags from the Copperbelt Province, Zambia. Mineral. Mag. 74, 581–600. doi: 10.1180/minmag.2010.074.4.581

[ref128] Wang M. X. Zhang Q. L. Yao S. J. (2015). A novel biosorbent formed of marine-derived penicillium janthinellum mycelial pellets for removing dyes from dye-containing wastewater. Chem. Eng. J. 259, 837–844. doi: 10.1016/j.cej.2014.08.003

[ref129] Watanabe T. (2016). Pictorial atlas of soil and seed fungi. Morphologies of Cultured Fungi and Key to Species.

[ref130] Wemedo S. Aleruchi O. (2020). Fungi in biodegradation of polycyclic aromatic hydrocarbons in oilfield wastewater. Acta Sci. Microbiol. 3, 220–224. doi: 10.31080/asmi.2020.03.0572

[ref131] Wickham H. (2009). Ggplot2: Elegant graphics for data analysis. Springer,New York, NY, USA,.

[ref132] Wong C. Tan L. T. Mujahid A. Lihan S. Wee J. L. S. Ting L. F.. (2018). Biosorption of copper by endophytic fungi isolated from Nepenthes ampullaria. Lett. Appl. Microbiol. 67, 384–391. doi: 10.1111/lam.13049 29998586

[ref133] Wood J. L. Liu W. Tang C. Franks A. E. (2016). Microorganisms in heavy metal bioremediation: strategies for applying microbial-community engineering to remediate soils. AIMS Bioeng. 3, 211–229. doi: 10.3934/bioeng.2016.2.211

[ref134] Wu B. Luo S. Luo H. Huang H. Xu F. Feng S.. (2021). Improved phytoremediation of heavy metal contaminated soils by Miscanthus floridulus under a varied rhizosphere ecological characteristic. Sci. Total Environ.:151995. doi: 10.1016/j.scitotenv.2021.151995 34856269

[ref135] Yin G. Zhang Y. Pennerman K. K. Wu G. Hua S. S. T. Yu J.. (2017). Characterization of blue mold penicillium species isolated from stored fruits using multiple highly conserved loci. J. Fungi 3:12. doi: 10.3390/jof3010012 PMC5715957 29371531

[ref136] Yu X. Zhan Q. (2020). “Phosphate-Mineralization Microbe Repairs Heavy Metal Ions That Formed Nanomaterials in Soil and Water,” in Nanomaterials - Toxicity, Human Health and Environment (IntechOpen).

[ref137] Zafar A. (2007). The growing relationship between China and sub-Saharan Africa: macroeconomic, trade, investment, and aid links. World Bank Res. Obs. 22, 103–130. doi: 10.1093/wbro/lkm001

[ref138] Zafar S. Aqil F. Ahmad I. (2007). Metal tolerance and biosorption potential of filamentous fungi isolated from metal contaminated agricultural soil. Bioresour. Technol. 98, 2557–2561. doi: 10.1016/j.biortech.2006.09.051 17113284

[ref139] Zhang X. Ding S. Lv H. Cui G. Yang M. Wang Y.. (2022). Microbial controls on heavy metals and nutrients simultaneous release in a seasonally stratified reservoir. Environ. Sci. Pollut. Res. 29, 1937–1948. doi: 10.1007/s11356-021-15776-4 34363164

